# Stress-induced ceramide generation and apoptosis via the phosphorylation and activation of nSMase1 by JNK signaling

**DOI:** 10.1038/cdd.2014.128

**Published:** 2014-08-29

**Authors:** T Yabu, H Shiba, Y Shibasaki, T Nakanishi, S Imamura, K Touhata, M Yamashita

**Affiliations:** 1 Nihon University, College of Bioresource Sciences, 1866 Kameino, Fujisawa, Kanagawa 252-0880, Japan; 2 Food Safety Assessment Research Group, National Research Institute of Fisheries Science, 12-4 Fukuura 2, Kanazawa-ku, Yokohama, Kanagawa 236-8648, Japan

## Abstract

Neutral sphingomyelinase (nSMase) activation in response to environmental stress or inflammatory cytokine stimuli generates the second messenger ceramide, which mediates the stress-induced apoptosis. However, the signaling pathways and activation mechanism underlying this process have yet to be elucidated. Here we show that the phosphorylation of nSMase1 (sphingomyelin phosphodiesterase 2, *SMPD2*) by c-Jun N-terminal kinase (JNK) signaling stimulates ceramide generation and apoptosis and provide evidence for a signaling mechanism that integrates stress- and cytokine-activated apoptosis in vertebrate cells. An nSMase1 was identified as a JNK substrate, and the phosphorylation site responsible for its effects on stress and cytokine induction was Ser-270. In zebrafish cells, the substitution of Ser-270 for alanine blocked the phosphorylation and activation of nSMase1, whereas the substitution of Ser-270 for negatively charged glutamic acid mimicked the effect of phosphorylation. The JNK inhibitor SP600125 blocked the phosphorylation and activation of nSMase1, which in turn blocked ceramide signaling and apoptosis. A variety of stress conditions, including heat shock, UV exposure, hydrogen peroxide treatment, and anti-Fas antibody stimulation, led to the phosphorylation of nSMase1, activated nSMase1, and induced ceramide generation and apoptosis in zebrafish embryonic ZE and human Jurkat T cells. In addition, the depletion of *MAPK8*/*9* or *SMPD2* by RNAi knockdown decreased ceramide generation and stress- and cytokine-induced apoptosis in Jurkat cells. Therefore the phosphorylation of nSMase1 is a pivotal step in JNK signaling, which leads to ceramide generation and apoptosis under stress conditions and in response to cytokine stimulation. nSMase1 has a common central role in ceramide signaling during the stress and cytokine responses and apoptosis.

The sphingomyelin pathway is initiated by the hydrolysis of sphingomyelin to generate the second messenger ceramide.^1^ Sphingomyelin hydrolysis is a major pathway for stress-induced ceramide generation. Neutral sphingomyelinase (nSMase) is activated by a variety of environmental stress conditions, such as heat shock,^1, 2, 3^ oxidative stress (hydrogen peroxide (H_2_O_2_), oxidized lipoproteins),^1^ ultraviolet (UV) radiation,^1^ chemotherapeutic agents,^4^ and *β*-amyloid peptides.^5, 6^ Cytokines, including tumor necrosis factor (TNF)-*α*,^7, 8, 9^ interleukin (IL)-1*β*,^10^ Fas ligand,^11^ and their associated proteins, also trigger the activation of nSMase.^12^ Membrane-bound Mg^2+^-dependent nSMase is considered to be a strong candidate for mediating the effects of stress and inflammatory cytokines on ceramide.^3^

Among the four vertebrate nSMases, nSMase1 (*SMPD2*) was the first to be cloned and is localized in the endoplasmic reticulum (ER) and Golgi apparatus.^13^ Several studies have focused on the potential signaling roles of nSMase1, and some reports have suggested that nSMase1 is important for ceramide generation in response to stress.^5, 6, 14, 15^ In addition, nSMase1 is responsible for heat-induced apoptosis in zebrafish embryonic cultured (ZE) cells, and a loss-of-function study showed a reduction in ceramide generation, caspase-3 activation, and apoptosis in zebrafish embryos.^16^ However, nSMase1-knockout mice showed no lipid storage diseases or abnormalities in sphingomyelin metabolism.^17^ Therefore, the molecular mechanisms by which nSMase1 is activated have yet to be elucidated.

Environmental stress and inflammatory cytokines^1, 18, 19, 20, 21, 22, 23, 24, 25, 26, 27^ stimulate stress-activated protein kinase (SAPK)/c-Jun N-terminal kinase (JNK) signaling, which involves the sequential activation of members of the mitogen-activated protein kinase (MAPK) family, including MAPK/ERK kinase kinase (MEKK)1/MAPK kinase (MKK)4, and/or SAPK/ERK kinase (SEK)1/MKK7, JNK, and c-jun. Both the JNK and sphingomyelin signaling pathways coordinately mediate the induction of apoptosis.^1^ However, possible crosstalk between the JNK and sphingomyelin signaling pathways has not yet been characterized. Previously, we used SDS-PAGE to determine that nSMase1 polypeptides migrated at higher molecular masses,^16^ suggesting that the sphingomyelin signaling pathway might cause the production of a chemically modified phosphorylated nSMase1, which is stimulated under stressed conditions in ZE cells.^16^ Here, we demonstrate that JNK signaling results in the phosphorylation of Ser-270 of nSMase1, which initiates ceramide generation and apoptosis. We also provide evidence for a signaling mechanism that integrates cytokine- and stress-activated apoptosis in vertebrate cells. We studied stress-induced ceramide generation in two cell types: ZE cells and human leukemia Jurkat T-lymphoid cells. Stress-induced apoptosis has been investigated in these systems previously.^16, 28^

## Results

### nSMase1 phosphorylation in response to stress stimuli

We used zebrafish ZE cells as a model to investigate the role of nSMase1, in addition to Jurkat T cells, which represent a more physiologically relevant human model. We demonstrated previously that zebrafish nSMase1 hydrolyzes sphingomyelin, induces apoptosis, and regulates ceramide generation via sphingomyelin metabolism in heat-stressed zebrafish ZE cells.^16^ Similar results were obtained in Jurkat T cells under various stressed conditions. The exposure of Jurkat T cells to heat shock (43 °C or 43.5 °C for 1 h), UV irradiation (1, 5, or 10 mJ/cm^2^ at 254 nm), H_2_O_2_ (0.5, 1, or 2 mM), and anti-Fas antibodies (25, 50, or 100 ng/ml) induced morphological and biochemical features typical of apoptosis, as determined using 4′,6-diamidino-2-phyenylidole (DAPI) staining (Supplementary Figure S1) and measurements of caspase-3 activity (Supplementary Figure S2). These effects were time- and dose-dependent.

**Figure 1:**
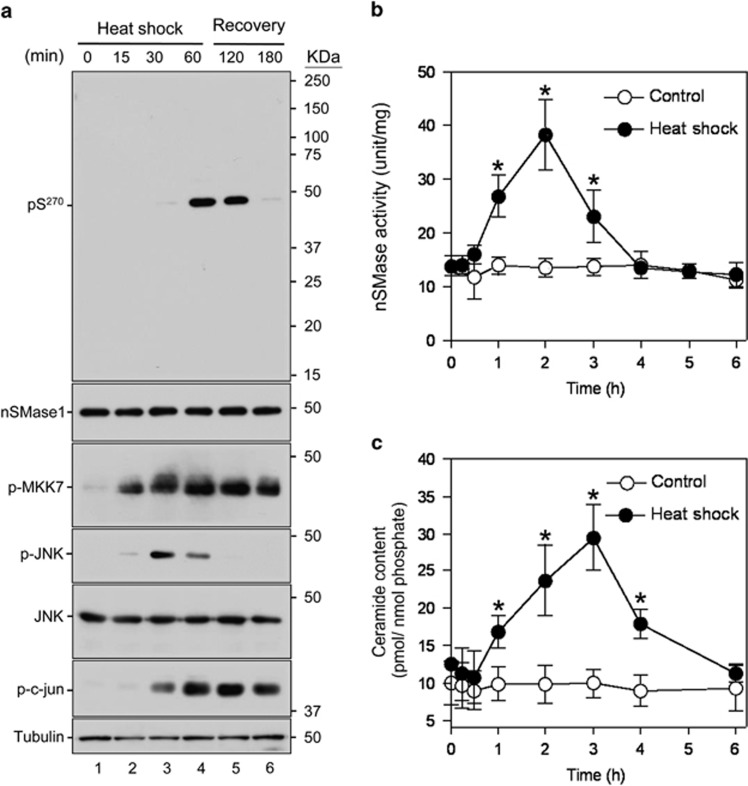
nSMase1 Ser-270 phosphorylation is induced by heat shock. (**a**) The Ser-270 phosphorylation of nSMase1 was induced in heat-shocked zebrafish cells. ZE cells were heat-shocked at 38 °C for 0, 15, 30, or 60 min and were then incubated at 25 °C for up to 2 h. The cell lysates were analyzed by western blotting with anti-phospho-Ser-270 nSMase1, anti-nSMase1, anti-phospho-MKK7, anti-phospho-JNK, anti-JNK, anti-phospho-c-jun, or anti-tubulin antibodies as indicated. Molecular weight markers are shown in kDa on the right. (**b**) Changes in the nSMase activity in response to heat-shock treatment in zebrafish cells. (**c**) Changes in ceramide generation in response to heat-shock treatment in zebrafish cells. The cells were heat-shocked at 38 °C for 1 h, and were then allowed to recover at 25 °C for the indicated times. The values represent the means of three independent experiments, and the error bars represent the S.D.s. *P<0.01 *versus* the control

We next assessed the activation mechanism of nSMase1 by testing the hypothesis that nSMase1 was phosphorylated by JNK or MAP kinases. A shifted band corresponding to phosphorylated nSMase1 detected by western blotting using an anti-nSMase1 antibody after ZE cells had been heat-shocked at 38 °C for 1 h (Supplementary Figure S3). Ser-270 in the conserved amino-acid sequence PXSDH near the catalytic center was predicted to be a potential phosphorylation site when the deduced amino-acid sequences of zebrafish (AB196165), human (NM_003080), and mouse (NM_009213) nSMase1 (Supplementary Figure S4) were aligned and analyzed using kinase-specific phosphorylation predictions.^29^ We successfully raised a specific antibody against the synthetic oligopeptide PFPY{phospho-S}DHEAMA, which specifically recognizes phosphorylated Ser-270 of nSMase1. When we heat-shocked ZE cells, Ser-270 phosphorylation was induced 60 min after heat shock at 38 °C and was maintained until 60 min after recovery at 25 °C (Figure 1a). The phosphorylation of the MAPK family members MKK7 and JNK, and the JNK downstream target c-jun, were also induced during the heat shock and recovery processes (Figure 1a). Heat shock also transiently increased nSMase activity in ZE cells (Figure 1b), which was accompanied by increased ceramide levels (Figure 1c). A ceramide accumulation peak was observed 3 h after heat shock at 38 °C for 1 h (Figure 1c). Because our previous study demonstrated a significant decrease in nSMase activity in response to antisense oligo knockdown in ZE cells under heat-shock conditions,^16^ we could assume that the stress-induced enzymatic activity was primarily associated with nSMase1 activation.

### Inhibition of nSMase1 phosphorylation by a JNK inhibitor in zebrafish cells

Environmental stresses stimulate ceramide generation through a process initiated by JNK signaling during stress-induced apoptosis.^1, 3^ We investigated whether the JNK or p38 mitogen-activated protein kinases (p38) stress signaling pathways affect nSMase activity by directly regulating core nSMase activation pathways during stress-induced apoptosis using specific inhibitors.^30^ Treating ZE cells with the specific JNK inhibitor SP600125 blocked both nSMase activation (Supplementary Figure S5a) and ceramide generation (Supplementary Figure S5b) under heat-shock conditions. However, treatment with the specific p38 inhibitor SB202190 had no effect (Supplementary Figure S5). SP600125 completely prevented the phosphorylation of nSMase1 and c-jun following heat-shock treatment of ZE cells (Supplementary Figure S5c). The phosphorylation of both MKK7 and JNK was detected in response to heat shock (Supplementary Figure S5c). Therefore, SP600125 blocks stress-activated nSMase and stress-induced ceramide formation, suggesting that the activated JNK cascade could block nSMase activation during the stress response.

Next, the specific interaction between phosphorylated nSMase1 and phosphorylated JNK was examined by immunoprecipitation. Phosphorylated JNK interacted with phosphorylated nSMase1, as determined by immunoprecipitation with an anti-phospho-JNK1/2 antibody (Supplementary Figure S6a) but only under heat-shock conditions (Supplementary Figure S6b). When ZE cells were pretreated with 10 *μ*M SP600125 for 1 h before heat-shock treatment, the interaction between nSMase1 and JNK was blocked. Therefore, we can conclude that nSMase1 is phosphorylated on Ser-270 under stress conditions via the direct binding of activated JNKs.

### *In vitro* phosphorylation of nSMase1 at Ser-270 by JNK1

**Figure 2:**
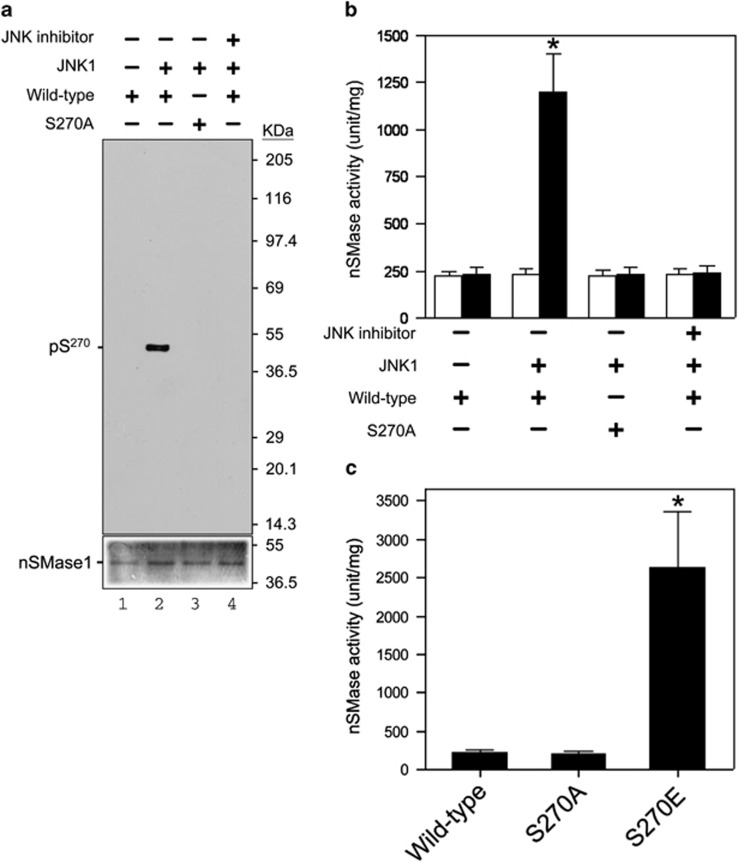
Phosphorylation and activation of nSMase1 by JNK *in vitro*. (**a**) The serine-270 phosphorylation of nSMase1 by JNK *in vitro*. Mouse JNK1 was used for an *in vitro* kinase assay using [*γ*-^32^ P]-ATP and either nSMase1 (wild-type) or mutant protein (S270A) as the substrate. The reactions were incubated at 30 °C for 30 min with or without 2 ng of mouse JNK1 and in the presence (+) or absence (−) of 10 *μ*M SP600125. Recombinant proteins from each reaction were separated on 10% SDS-polyacrylamide gels and transferred to PVDF membranes. Phosphorylated nSMase1 was detected by autoradiography (upper panel). The levels of substrate protein present in each reaction was determined using Coomassie Brilliant Blue R-250 staining (lower panel). (**b**) The effect of phosphorylation on the activation of nSMase1. The nSMase activity in each column is shown. After the *in vitro* kinase assay, the postreaction mixture was analyzed in an nSMase enzymatic assay using C_6_-NBD-sphingomyelin (black columns). White columns indicate the basal enzyme activity before the kinase assay. The basal enzyme activities of the recombinant nSMase1 (wild-type) and nSMase1 mutant (S270A) were 22.4±0.48 and 22.6±0.35 *μ*mol/mg/h, respectively. Column 1, nSMase1 wild-type; column 2, nSMase1 treated with JNK; column 3, nSMase1 mutant (S270A) treated with JNK; column 4, nSMase1 treated with JNK in the presence of a JNK inhibitor. The enzyme activity after JNK treatment (black column 2) was 120.1±21.4 *μ*mol/mg/h. Each value represents the mean of three independent experiments, and the error bars represent the S.D.s. *P<0.01 *versus* the wild-type. (**c**) Activities of the wild-type and S270A and S270E nSMase1 mutants. The nSMase activities of the recombinant proteins were determined using C6-NBD-sphingomyelin. Column 1, recombinant nSMase1 (wild-type); column 2, S270A mutant (S270A); column 3, S270E mutant (S270E). The activity of the S270E mutant (column 3) was 237.8±81.7 *μ*mol/mg/h. Each value represents the mean of three independent experiments, and the error bars represent the S.D.s. *P<0.01 *versus* basal enzyme activity (white column 2)

We next investigated which protein kinase phosphorylates Ser-270 of nSMase1 *in vitro*. To determine the involvement of JNK1, we prepared two recombinant protein versions of zebrafish nSMase1, a wild-type nSMase1 (wild-type) and a mutant protein lacking Ser-270 (S270A), as substrates for the JNK1 kinase reaction using ^32^P-radiolabeled ATP.^16^ In the autoradiogram shown in Figure 2a, wild-type nSMase1 was phosphorylated by JNK1 kinase (lane 2), whereas the S270A mutant (lane 3), wild-type alone (lane 1), and wild-type pulsed with SP600125 were not phosphorylated (Figure 2a). Therefore, Ser-270 of nSMase1 was phosphorylated by JNK1.

The nSMase activity of wild-type nSMase1 was enhanced by about four-fold by JNK1 compared with basal levels wild-type nSMase1 activity. In contrast, the nSMase activity of the S270A mutant was not enhanced (column 2) (Figure 2b). The enzyme activity of the S270A mutant (column 3), wild-type alone (column 1), and wild-type nSMase1 pulsed with SP600125 (column 4) was also unaffected (Figure 2b). Therefore, Ser-270 of nSMase1 was a key phosphorylation site in nSMase1, as its phosphorylation induced enzyme activation *in vitro*.

We next investigated whether recombinant nSMase1 in which Ser-270 was replaced with negatively charged glutamic acid exhibited enhanced nSMase activity *in vitro*. We purified three recombinant proteins, wild-type nSMase1 (wild-type) and two mutant proteins lacking the Ser-270 phosphorylation site (S270E and S270A) and then measured their nSMase activities. The activity of the S270E mutant was ∼11-fold greater than the wild-type enzyme (Figure 2c). Therefore, substituting Ser-270 with negatively charged glutamic acid mimicked the effects of phosphorylation.

### The overexpression of nSMase1 and its mutants stimulates ceramide generation and apoptosis in zebrafish cells

**Figure 3:**
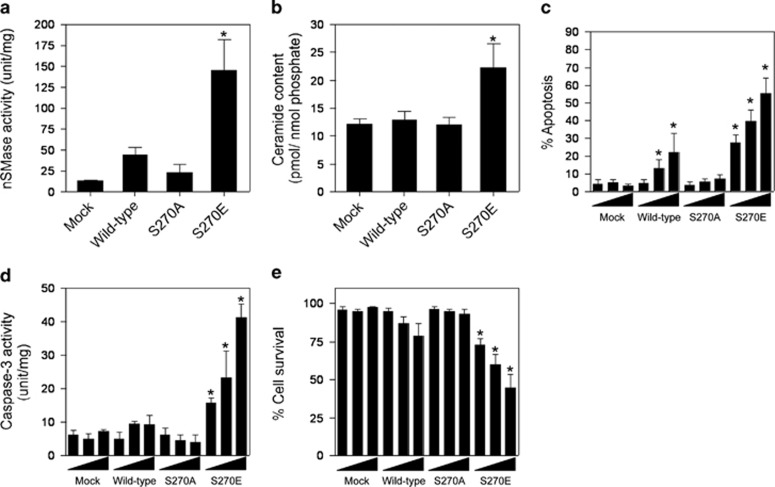
Induction of ceramide generation and apoptosis by the overexpression of mutant S270E in zebrafish ZE cells. (**a**) The nSMase activities of nSMase1 transfectants. ZE cells were transiently transfected with three different doses (1, 2, or 3 *μ*g) of mock, FLAG-tagged wild-type, S270A, or S270E SMase1 vector DNA constructs and cultured for 24 h. nSMase activity was measured using C_6_-NBD-sphingomyelin as a substrate. Column 1, mock; column 2, wild-type; column 3, S270A; column 4, S270E. (**b**) The ceramide content in nSMase1 transfectants. Cellular lipids were extracted from the nSMase1 transfectants, and the levels of ceramide were quantified using a diacylglycerol kinase assay after thin layer chromatography separation. Column 1, mock; column 2, wild-type; column 3, S270A; column 4, S270E. (**c**) Apoptosis in nSMase1-transfected cells. ZE cells were transfected with three different doses (1, 2, or 3 *μ*g) of mock, FLAG-tagged wild-type, S270A, or S270E SMase1 vector DNA constructs, cultured for 24 h, and stained with DAPI to quantify apoptosis. (**d**) Caspase-3 activity in nSMase1 transfectants. Caspase-3 activation was assessed by measuring Ac-DEVD-MCA hydrolysis. (**e**) The viability of nSMase1-transfected cells, as determined using Trypan Blue dye exclusion. (**f**) Apoptotic cells in the nSMase1 transfectants after heat shock. ZE cells were transfected with three different doses (1, 2, or 3 *μ*g) of nSMase1 constructs per dish and cultured for 24 h. After heat shock at 38 °C for 1 h, the cells were incubated at 25 °C for up to 23 h, and all apoptotic cells were identified using DAPI staining. Each value represents the mean of three independent experiments, and the error bars represent the S.D.s. *P<0.01 *versus* mock

When we transiently overexpressed the mock, wild-type, S270A, and S270E mutants of nSMase1 in ZE cells, cells overexpressing S270E exhibited higher nSMase activity (Figure 3a) and ceramide levels (Figure 3b) than those overexpressing the mock mutants. ZE cells were transfected with 1, 2, or 3 *μ*g of mock, wild-type, S270A, or S270E mutant nSMase1 DNA. Apoptosis was induced in cells transfected with the wild-type and the S270E mutant in a dose-dependent manner, whereas no effect was observed in cells transfected with mock and the S270A mutant (Figure 3c). Similarly, caspase-3 activation was induced in cells transfected with the S270E mutant in a dose-dependent manner, whereas no effect was observed in cells transfected with mock, wild-type, and the S270A mutant (Figure 3d). Viability decreased in cells transfected with the S270E mutant but not in those transfected with mock, wild-type, or the S270A mutant (Figure 3e). The ceramide content of wild-type transfected cells did not increase (Figure 3b), whereas nSMase activity (Figure 3a) and apoptosis (Figure 3c) were both elevated, indicating that the overexpressed wild-type construct had a weak apoptosis-inducing effect. Substitution of Ser-270 with negatively charged glutamic acid mimicked the effect of phosphorylation, leading to nSMase1 activation and subsequent induction of ceramide generation and apoptosis.

We also investigated whether the S270A mutant could block the endogenous phosphorylation of nSMase1 under heat-stress conditions in ZE cells. We treated the S270A, wild-type, and mock transfectants with heat shock and assessed nSMase1 phosphorylation. The overexpression of wild-type nSMase1 and the S270A mutant was examined by western blotting. Signals for protein bands were higher in the wild-type and S270A mutant transfectants (lanes 3–6) than in the mock transfectant (lanes 1 and 2) (Supplementary Figure S7a). Phosphorylation at Ser-270 was induced in the mock by heat shock (lane 2) and increased significantly in the wild-type (lane 6) transfectants (Supplementary Figure S7a). In contrast, expression of the S270A mutant blocked the endogenous phosphorylation of nSMase1 (lane 4, Supplementary Figure S7a). Although the overexpression of the wild-type construct induced apoptosis weakly (Figure 3c), the phosphorylation at Ser-270 was not detected (lane 5, Supplementary Figure S7a). The phosphorylation of MKK7, JNK, and c-jun was induced by heat shock in all cell transfectants (Supplementary Figure S7a). Under heat-shock conditions, nSMase activity (Supplementary Figure S7b), ceramide levels (Supplementary Figure S7c), and number of apoptotic cells (Supplementary Figure S7d) were elevated in wild-type transfectants but were repressed in S270A-transfected cells. Therefore, the S270A mutant functions as a dominant-negative (DN) suppressor of stress-activated nSMase1, ceramide formation, and apoptosis in ZE cells.

**Figure 4:**
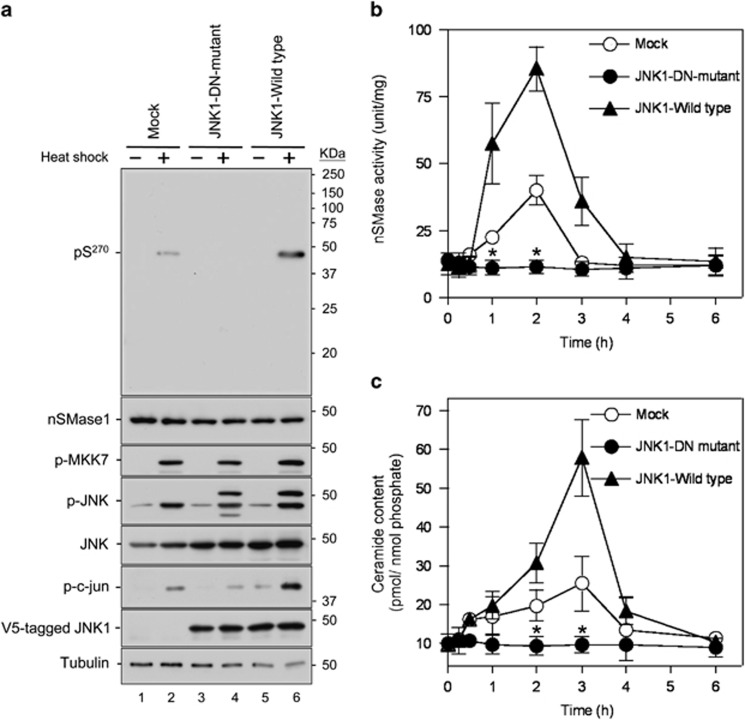
Activation and phosphorylation of nSMase1 after the expression of a JNK DN mutant in heat-stressed zebrafish cells. (**a**) Detection of nSMase1 phosphorylation by the overexpression of JNK and its DN mutant (K55R). Mock cells (lanes 1 and 2), JNK1-DN (DN) mutant cells (lanes 3 and 4), and JNK1 wild-type cells (lanes 5 and 6) were either unstressed (normal growth, −) at 25 °C for 60 min, or heat-shocked (+) at 38 °C for 60 min. They were then analyzed by western blotting with antibodies against phosphorylated nSMase1, nSMase1, phosphorylated MKK7, phosphorylated JNK, JNK, phosphorylated c-jun, V5-tagged JNK1 wild-type, V5-tagged JNK1-DN mutant, and tubulin. Molecular weight markers are shown in kDa on the right. (**b**) Changes in nSMase activity. Mock, JNK1-DN, and JNK1 wild-type cells were heat-shocked at 38 °C for 0, 15, 30, or 60 min, allowed to recover at 25 °C for up to 5 h, and then harvested at the indicated times. nSMase activity was measured using C_6_-NBD-sphingomyelin as a substrate. Values represent the means of three independent experiments, and the error bars represent the S.D.s. *P<0.01 *versus* the mock control. (**c**) Changes in ceramide content. The ceramide content was measured using the diacylglycerol kinase assay. Values represent the means of three independent experiments, and error bars represent S.D.s. *P<0.01 *versus* the mock control

Because JNK1 homodimerization is required for its activation,^31, 32^ we investigated the effect of a DN JNK1 mutant on the stress-induced activation of nSMase1. We constructed a kinase-dead JNK1- DN mutant K55R, in which lysine-55, which is critical for phosphotransfer in the ATP-binding motif, is mutated to arginine and generated stable transfectants in ZE cells. JNK1 expression increased the phosphorylation of nSMase1 and c-jun following heat shock (lane 6; Figure 4a). In contrast, expression of the JNK1-DN-mutant inhibited the phosphorylation of nSMase1 and c-jun (lane 4; Figure 4a). Phosphorylation of nSMase1 in response to heat shock was also detected in the control cells with the mock vector (lane 2; Figure 4a). The phosphorylation of MKK7 and JNK was detected in all three transfectants (mock, JNK1-DN-mutant, and JNK1-wild type) following heat shock (Figure 4a). In the JNK1-DN-mutant cells, nSMase activity (Figure 4b) and ceramide levels (Figure 4c) decreased after heat shock. We can conclude that the S270E nSMase1 mutant mimicked the phosphorylated enzyme, leading to induced ceramide generation and apoptosis. In contrast, the repression of JNK signaling by overexpression of the S270A mutant or the JNK1-DN-mutant negatively regulated nSMase1 activation and ceramide signaling in zebrafish cells.

### Phosphorylation of Ser-270 of nSMase1 by stress and Fas stimuli in Jurkat T cells

**Figure 5:**
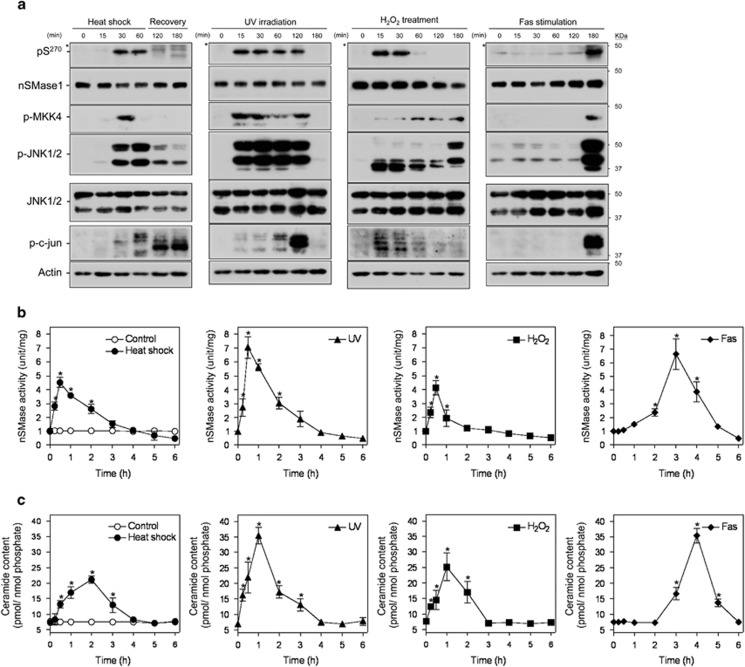
Stress-induced nSMase1 Ser-270 phosphorylation, nSMase activation, and ceramide generation in human Jurkat T cells. (**a**) The phosphorylation of Ser-270 nSMase1 was detected in Jurkat T cells. Cells were heat-shocked at 43 °C for 0, 15, 30, or 60 min and were then recovered at 37 °C for up to 2 h. For UV irradiation, the cells were irradiated by 254 nm UV at 5 mJ/cm^2^. For H_2_O_2_ treatments, cells were incubated in the presence of 1 mM H_2_O_2_. For Fas stimulation, cells were treated with 50 ng/ml of anti-Fas antibody and incubated at 37 °C for 0, 15, 30, 60, 120, or 180 min. The cell lysates were analyzed by western blotting with anti-phospho-Ser-270 nSMase1, anti-nSMase1, anti-phospho-MKK7, anti-phospho-MKK4, anti-phospho-JNK, anti-JNK, anti-phospho-c-Jun, or anti-actin antibodies, as indicated. Molecular weight markers are shown in kDa on the right. Asterisks indicate nonspecific signals. (**b**) The effect of heat shock, ultraviolet light (UV), hydrogen peroxide (H_2_O_2_), and anti-Fas antibody (Fas) on nSMase activity in Jurkat T cells. (**c**) The effect of heat shock, UV, H_2_O_2_, and Fas on ceramide generation in Jurkat T cells. Values represent the means of three independent experiments, and the error bars represent the S.D.s. *P<0.01 *versus* the control

Based on the findings of the phosphorylated activation of nSMase1 in zebrafish cells, we next investigated the significance of nSMase1 phosphorylation on ceramide signaling and apoptosis in human cells. The anti-phospho (Ser-270)-nSMase1 antibody detected phosphorylated nSMase1 in Jurkat T cells following heat shock at 43 °C for 30–60 min, 5 mJ/cm^2^ UV irradiation for 15–120 min, 1 mM H_2_O_2_ treatment for 15–30 min, and stimulation with 50 ng/ml anti-Fas antibody for 180 min (Figure 5a). MKK4, JNK1/2, and c-jun were also phosphorylated in response to these conditions (Figure 5a). The exposure of Jurkat T cells to heat shock at 43 °C for 1 h, UV irradiation at 5 mJ/cm^2^ and 254 nm, 1 mM H_2_O_2_, and anti-Fas antibody (50 ng/ml) transiently increased nSMase activity (Figure 5b), with a concurrent increase in ceramide levels (Figure 5c). Ceramide accumulation peaks were observed 2 h after heat shock at 43 °C for 1 h, 1 h after UV irradiation, 1 h after H_2_O_2_, and 4 h after anti-Fas antibody treatment (Figure 5c).

Overexpression of the nSMase1 S270E mutant elicited nSMase activity in ZE cells (Figure 3). To confirm that the S270E mutant was able to induce nSMase activity in human cells, the mock, wild-type, S270A, and S270E mutant constructs were transiently transfected in Jurkat T cells. Apoptosis and caspase-3 activation were induced in cells transfected with the wild-type and the S270E mutant in a dose-dependent manner, whereas no effect occurred in cells transfected with the mock and S270A mutant (Supplementary Figures S8c and d). Cells expressing the S270E mutant exhibited higher nSMase activities (Supplementary Figure S8a) and ceramide levels (Supplementary Figure S8b) than those transfected with the mock construct. Viability decreased in cells transfected with the S270E mutant but not in those transfected with the mock, wild-type, or S270A mutant (Supplementary Figure S8e). Therefore, Ser-270 phosphorylation is also induced by stress and Fas stimuli in Jurkat T cells. Furthermore, ceramide generation, caspase-3 activation, and apoptosis induction are associated with phosphorylated activation of nSMase1. In addition, the S270E mutant, which was characterized as an active form of nSMase1 in zebrafish cells, also mimicked the phosphorylated enzyme, leading to induced ceramide generation and apoptosis in Jurkat T cells.

### JNK signaling stimulates nSMase 1 phosphorylation in Jurkat T cells

To confirm the JNK1/2-dependent mechanisms of nSMase1 phosphorylation in human cells, JNK1 and JNK2, which are encoded by *MAPK8* and *MAPK9*,^25^ were knocked down using *MAPK8* and/or *MAPK9* RNAi. The expression of total JNK1/2 was partially diminished by *MAPK8* or *MAPK9* RNAi and was blocked completely by the combination of *MAPK8* and *MAPK9* RNAi (Supplementary Figure S9).

**Figure 6:**
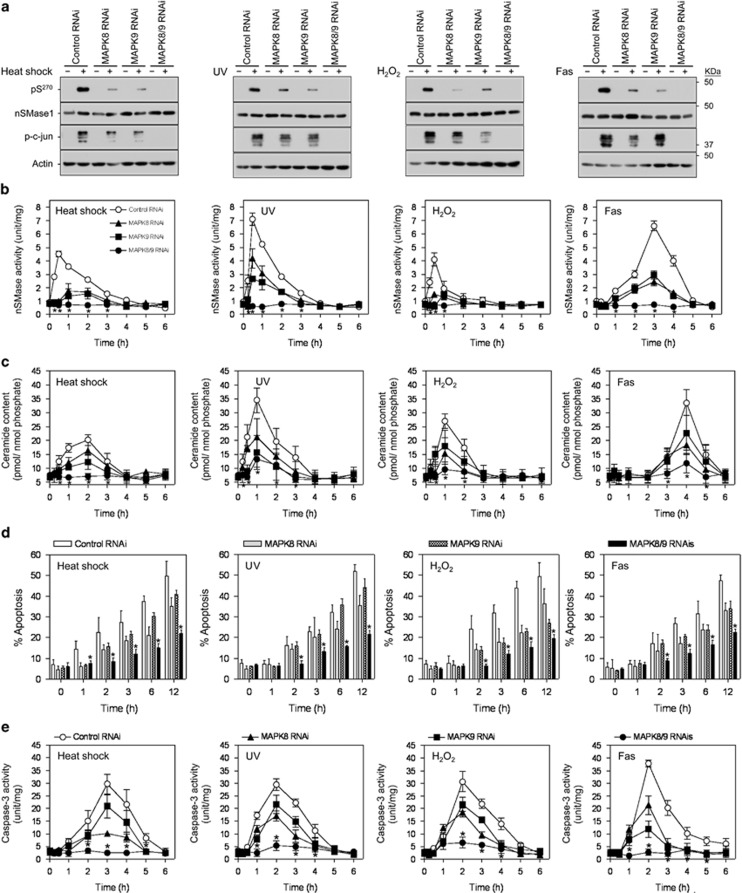
An essential role for *MAPK8* and *MAPK9* in nSMase1 activation and ceramide generation in human Jurkat T cells in a loss-of-function study. (**a**) Detection of phosphorylated-nSMase1 by western blotting after the RNAi-mediated knockdown of *MAPK8* and/or *MAPK9* in Jurkat T cells. Jurkat T cells were transfected with control, *MAPK8*, and/or *MAPK9* RNAi and then heat-shocked at 43 °C for 30 min, UV-irradiated at 254 nm and 5 mJ/cm^2^ for 30 min, treated with 1 mM H_2_O_2_ for 30 min, or stimulated with 50 ng/ml of anti-Fas antibody for 3 h. Cell lysates were harvested and analyzed by western blotting with anti-phospho-nSMase1, anti-nSMase1, anti-JNK, anti-phospho-c-jun, or anti-actin antibodies. Molecular weight markers are shown in kDa on the right. *MAPK8* and *MAPK9* are the human genes encoding JNK1 and JNK2, respectively. (**b**) nSMase activation. RNAi-transfected cell lines were stressed as in panel (**a**) and then incubated at 37 °C for up to 5 h. (**c**) Ceramide generation. (**d**) Effect of loss of function of *MAPK8* and *MAPK9* on the induction of apoptosis. Twenty-four hours after RNAi transfection, apoptosis was quantified in the *MAPK8-* and/or *MAPK9-*knockdown cells using DAPI staining after stress treatment as described in panel (**a**). (**e**) Effect of loss of function of *MAPK8* and *MAPK9* on apoptosis induction assessed using a caspase-3 assay. Each value represents the mean of three independent experiments, and the error bars represent the S.D.s. *P<0.01 *versus* control RNAi

Phosphorylation of nSMase1 at Ser-270 was induced in cells transfected with control RNAi under stressed conditions such as heat shock at 43 °C for 30 min (Figure 6a), UV irradiation with 5 mJ/cm^2^ for 30 min (Figure 6a), 1 mM H_2_O_2_ treatment for 30 min (Figure 6a), and 50 ng/ml of anti-Fas antibody for 180 min (Figure 6a). In contrast, the phosphorylation of nSMase1 at Ser-270 and c-jun was blocked completely in JNK1/2-deficent cells treated with both *MAPK8* and *MAPK9* RNAi (Figure 6a). The phosphorylation of nSMase1 and c-jun was inhibited partially by transfection with RNAi for *MAPK8* or *MAPK9* (Figure 6a). Stress-induced nSMase activation (Figure 6b) and ceramide generation (Figure 6c) were also reduced in JNK1/2-deficent cells treated with RNAi against *MAPK8* and/or *MAPK9* compared with cells treated with control RNAi. Therefore, nSMase1 is phosphorylated at Ser-270 by JNK1/2 protein kinases in human cells. In addition, exposing *MAPK8-* and/or *MAPK9-*knocked-down cells to heat shock at 43.5 °C for 1 h, UV irradiation at 5 mJ/cm^2^ and 254 nm, 1 mM H_2_O_2_, and 50 ng/ml anti-Fas antibody induced morphological and biochemical features typical of apoptosis, as determined using DAPI staining (Figure 6d) and a caspase-3 activity assay (Figure 6e). These effects were JNK1/2 depletion- and time-dependent.

In Jurkat T cells, phosphorylated nSMase1 was detected by immunoprecipitation with anti-phospho-JNK1/2 antibodies after stress stimuli, including heat shock (lane 3), UV irradiation (lane 4), H_2_O_2_ treatment (lane 5), and anti-Fas antibody stimulation (lane 6) but not in unstressed cells (lane 2) (Supplementary Figure S10a). Immunoprecipitation with anti-JNK1/2 antibodies suggests that JNK1/2 interacted with nSMase1 in response to these stress stimuli but not under control conditions (Supplementary Figure S10b).

To confirm the effect of JNK signaling on the phosphorylation of nSMase1 at Ser-270 under stress conditions in Jurkat T cells, MKK4 and MKK7, which are encoded by *MAP2K4* and *MAP2K7,* respectively, were knocked down using *MAP2K4* and/or *MAP2K7* RNAi. The expression of MKK4 and MKK7 were diminished by *MAP2K4* and/or *MAP2K7* RNAi and was blocked completely by the combination of both RNAis (Supplementary Figure S11a). The phosphorylation of Ser-270 of nSMase1 and JNK1/2 was induced in cells transfected with control RNAi under stressed conditions, such as UV irradiation and treatment with 1 mM H_2_O_2_ (Supplementary Figure S11b). In contrast, the phosphorylation of Ser-270 of nSMase1 and c-jun was blocked completely in MKK4/7-deficent cells treated with RNAi against both *MAP2K4* and *MAP2K7* under stressed conditions such as UV irradiation and H_2_O_2_ treatment (Supplementary Figure S11b). Therefore, the phosphorylated activation of nSMase1 is mediated by binding of phosphorylated JNK1/2 in response to various stress stimuli and Fas stimulation.

### Essential role of nSMase1 in stress-induced ceramide formation and apoptosis

**Figure 7:**
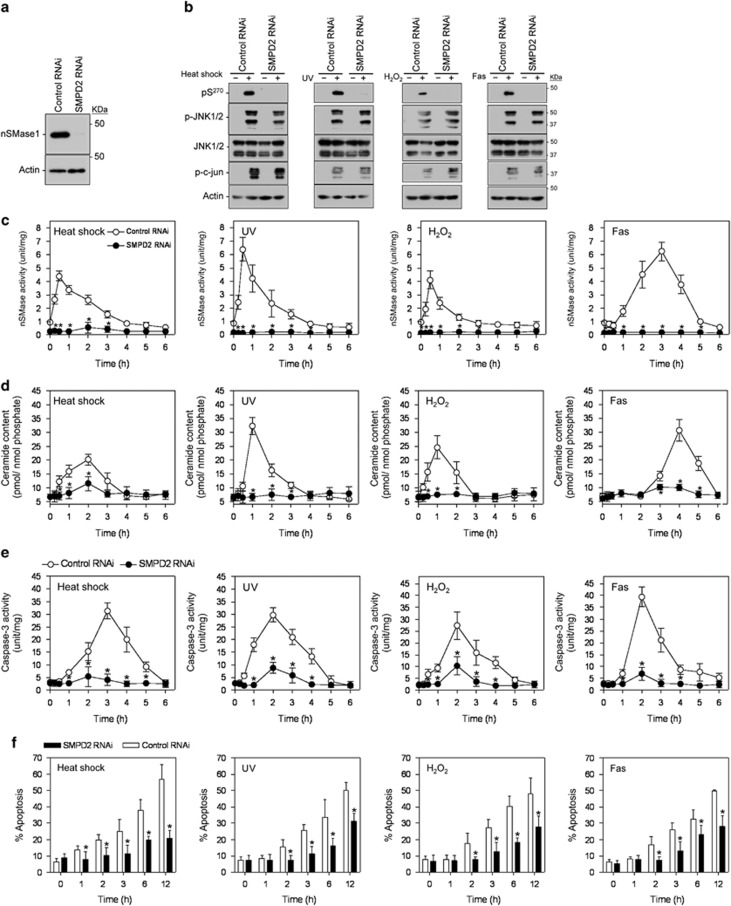
Essential role of nSMase1 in stress-induced ceramide generation in human Jurkat T cells in a loss-of-function study. (**a**) Knockdown of *SMPD2* expression in human Jurkat T cells. (**b**) Detection of phosphorylated-nSMase1 by western blotting. The cells were transfected with control or *SMPD2* RNAi and cultured for 24 h. The transfected cells were stressed using heat shock at 43 °C for 30 min, UV-irradiated at 5 mJ/cm^2^ for 30 min, 1 mM H_2_O_2_ treatment for 30 min, and stimulation with 50 ng/ml anti-Fas antibody for 3 h. Cell lysates were harvested and analyzed by western blotting with anti-phospho-nSMase1, anti-nSMase1, anti-phospho-JNK, anti-JNK, anti-phospho-c-jun, or anti-actin antibodies. Molecular weight markers are shown in kDa on the right. (**c**) nSMase1 activation by *SMPD2* knockdown. The cell lines were heat-shocked at 43 °C for 0, 15, 30, or 60 min, irradiated at 5 mJ/cm^2^ UV for 0, 15, 30, or 60 min, treated with 1 mM H_2_O_2_ for 0, 15, 30, or 60 min, and stimulated with 50 ng/ml of anti-Fas antibody for 0, 15, 30, or 60 min. The cells were then incubated at 37 °C for up to 5 h. (**d**) Ceramide generation after *SMPD2* knockdown. (**e** and **f**) Effect of *SMPD2* knockdown on the induction of apoptosis in human Jurkat T cells. Each value represents the mean of three independent experiments, and the error bars represent the S.D.s. *P<0.01 *versus* the control

We demonstrated previously that the activation of nSMase1 was essential for stress-induced ceramide generation and apoptosis in zebrafish cells.^16^ To assess whether this pathway was conserved in human cells, the *SMPD2* gene, which encodes nSMase1, was knocked down in Jurkat T cells using RNAi, and stress-induced ceramide formation and apoptosis were measured. When the expression of *SMPD2* was knocked down, the expression of nSMase1 completely disappeared (Figure 7a). The phosphorylation of nSMase1 at Ser-270 was induced in cells transfected with control RNAi under stressed conditions and anti-Fas stimuli but not in *SMPD2-*knockdown cells (Figure 7b). Phosphorylated JNK1/2 and c-jun were produced under stressed conditions and after anti-Fas stimulation in both control and *SMPD2-*knockdown cells (Figure 7b). nSMase activity (Figure 7c) and ceramide levels (Figure 7d) were induced by stress and Fas stimuli in cells treated with control, but not *SMPD2* RNAi, although ceramide levels increased slightly in the *SMPD2-*knockdown cells 1 h after heat shock. As RNAi against nSMase1 resulted in a significant decrease in nSMase activity following the stress stimulus, we can conclude that the increase in enzymatic activity in response to stress stimuli is primarily a consequence of nSMase1 activation. Under non-stressed conditions, ceramide levels were maintained at ∼7 pmol/nmol phosphate in both the control and *SMPD2-*knockdown cells. Exposure of *SMPD2-*knockdown cells to heat shock at 43 °C for 1 h, UV irradiation at 5 mJ/cm^2^ and 254 nm, 1 mM H_2_O_2_, and 50 ng/ml anti-Fas antibody induced the biochemical and morphological features typical of apoptosis, as confirmed by caspase-3 activity measurements (Figure 7e) and DAPI staining (Figure 7f). The observed effects were *SMPD2*-depletion- and time-dependent.

We also confirmed that pretreating Jurkat T cells with 40 *μ*M caspase inhibitor (carboxybenzyl-VAD-fluoromethyl ketone (z-VAD-fmk)) for 1 h prevented stress-induced apoptosis under conditions such as heat shock at 43 °C for 1 h, UV irradiation at 5 mJ/cm^2^, 1 mM H_2_O_2_, and treatment with 50 ng/ml of anti-Fas antibody (Supplementary Figure S12).

Cells expressing the S270E mutant exhibited nSMase activity, ceramide generation, and apoptosis in Jurkat cells (Supplementary Figures S8a–c). To confirm whether phosphorylated SMase1 mediates caspase-dependent apoptosis, we examined the effects of z-VAD-fmk on the induction of apoptosis and cell viability of nSMase1 mutant transfectants. After treatment with z-VAD-fmk for 1 h prior to transfection, the mock, wild-type, H272A, S270A, and S270E mutant constructs were transiently transfected into Jurkat T cells. Induction of apoptosis and reduction of cell viability were blocked in the transfected cells with the S270E mutant but not in those with the mock, H272A and S270A mutants (Supplementary Figure S13). We can therefore conclude that ceramide generation by phosphorylated SMase1 or the constitutively active mutant was found to be essential for caspase-3 activation and induction of apoptosis in Jurkat T cells.

### Subcellular localization of phosphorylated nSMase1

nSMase1 is predominantly localized to the ER and Golgi apparatus.^13, 14, 16^ We examined the subcellular localization of nSMase1 by ultracentrifugation, and data revealed that nSMase1 was present in the microsomal fractions with the cell membrane marker pan-cadherin but not in the cytosolic fraction with the cytosolic marker aldolase (Supplementary Figure S14). The levels of phosphorylated nSMase1 increased in the microsomal fractions under stressed conditions of heat shock, UV irradiation, H_2_O_2_ treatment, and Fas stimulation (Supplementary Figure S14). Because nSMase1 was localized to the nuclear matrix in rat cells,^33^ we also assessed the nuclear localization of nSMase1 in Jurkat T cells. nSMase1 was detected in nuclear fractions with the nuclear marker histone H2A. Phosphorylated nSMase1 was induced by stress stimuli, including heat shock, UV irradiation, H_2_O_2_ treatment, and anti-Fas antibody stimuli (Supplementary Figure S15). As shown in Supplementary Figure S16, we also examined the subcellular localization of phosphorylated nSMase1 under non-stress conditions (Control, Supplementary Figures S16a and f) and stress conditions, such as heat shock at 43 °C for 30 min (Heat shock, Supplementary Figures S16b and g), UV irradiation at 5 mJ/cm^2^ (UV, Supplementary Figures S16c and h), H_2_O_2_ treatment at 1 mM for 30 min (H_2_O_2_, Supplementary Figures S16d and i), and anti-Fas antibody treatment at 50 ng/ml for 3 h (Fas, Supplementary Figures S16e and j) via immunocytochemical staining. Phosphorylated nSMase1 co-localized with the ER marker calnexin (Supplementary Figures S16a–e) and the Golgi apparatus marker 58-k Golgi protein (Supplementary Figures S16f–j) in Jurkat T cells. In the control cells under non-stress conditions, weak signals of phosphorylated nSMase1 were detected in the nucleus, indicating that phosphorylated nSMase1 is present in the nucleus under non-stress conditions. Our findings based on subcellular fractionation and immunocytochemical staining indicate that nSMase1 and its phosphorylated form localized primarily to the Golgi apparatus, ER, and nuclear fractions in Jurkat T cells under stress conditions and upon Fas stimulation.

## Discussion

This study revealed that nSMase1 is a substrate of the JNK signaling pathway and that it was phosphorylated at Ser-270 in response to stress and Fas stimulation. The specific signal for phosphorylated nSMase1 as a result of JNK signaling was detected using antibodies against nSMase1 that was phosphorylated on Ser-270. This phosphorylation event activated the enzyme and induced ceramide generation and apoptosis in both zebrafish ZE cells and human Jurkat T cells.

In zebrafish cells, substituting Ser-270 for alanine blocked the phosphorylated activation of nSMase1, whereas substitution for negatively charged glutamic acid mimicked the effect of phosphorylation. The constitutively active S270E mutant of nSMase1 induced ceramide generation and apoptosis. In contrast, repression of JNK signaling by overexpression of the S270A mutant or the JNK1-DN-mutant negatively regulated nSMase1 activation and ceramide signaling in zebrafish cells. Overexpression of the wild-type construct had a weak effect on apoptosis induction, with no detectable phosphorylation at Ser-270. Although the JNK inhibitor SP600125 has been reported to inhibit many different kinases,^34^ SP600125, but not the specific p38 inhibitor SB202190, blocked the phosphorylation and activation of nSMase1, which in turn blocked ceramide signaling and apoptosis.

**Figure 8:**
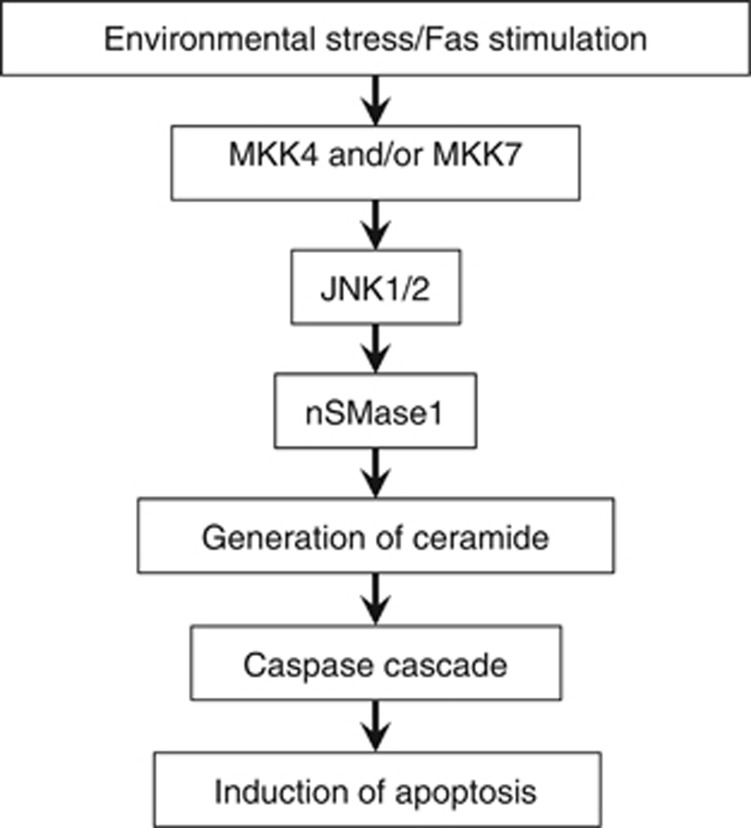
Diagram connecting nSMase1 and the JNK signaling pathway

In Jurkat T cells, a variety of stress conditions, including heat shock, UV irradiation, H_2_O_2_ treatment, and Fas stimulation, led to the phosphorylation and activation of nSMase1, which induced ceramide generation and apoptosis. The overexpression of the wild-type and S270E mutant also induced apoptosis and caspase-3 activation in Jurkat T cells. Viability decreased in cells transfected with the S270E mutant but not in those transfected with mock, wild-type, or S270A mutant. In contrast, the depletion of *MAPK8*/*9* or *SMPD2* using RNAi decreased ceramide generation and stress- and cytokine-induced apoptosis. The effects of silencing *MAP2K4/7*, *MAPK8*/*9*, or *SMPD2* on the phosphorylation of Ser-270 of nSMase1 were confirmed after exposure to stress stimuli. The caspase inhibitor z-VAD-fmk prevented apoptosis as induced by JNK-dependent nSMase1 phosphorylation on Ser-270. Therefore, the phosphorylation of nSMase1 is a pivotal step in JNK signaling that leads to ceramide generation and apoptosis under stress conditions and in response to cytokine stimulation. nSMase1 has a common central role in ceramide signaling in both stress and cytokine responses and apoptosis. Previous studies have reported both early and late activation of nSMases in different cell types.^3, 35^ The present findings indicate that nSMase1 is responsible for ceramide signaling and apoptosis induction in response to JNK signaling during short time course of stress and cytokine stimulation. As summarized in Figure 8, environmental stresses (e.g., heat shock, UV exposure, H_2_O_2_ treatment, and *γ*-irradiation) and inflammatory cytokines (e.g., TNF-*α*, IL-1*β*, and Fas ligand) might stimulate nSMase1 phosphorylation, which in turn is regulated by JNK1/2-dependent signal transduction.

nSMase1 is ubiquitously expressed in animal cells and tissues.^13^ The present study revealed that JNK-mediated nSMase1 activation and ceramide generation are responsible for various physiological and pathological regulatory effects in both zebrafish and human cells. We demonstrated previously that zebrafish nSMase1 hydrolyzes sphingomyelin, reduces lyso-platelet-activating factor (lyso-PAF) phospholipase C activity, induces apoptosis, and regulates ceramide generation via sphingomyelin metabolism under stress conditions.^16^ The present study confirms the significance of nSMase1 in ceramide generation and apoptosis induction in response to JNK signaling. However, the regulation and cellular functions of mammalian nSMase1 during ceramide generation and apoptosis induction have not yet been elucidated. In T-cell hybridoma 3DO cells, nSMase1 knockdown suppressed the ceramide-mediated apoptosis that was triggered by T-cell receptor ligation.^15^ The knockdown of nSMase inhibited amyloid peptide-induced ceramide generation and apoptosis in rat oligodendrocytes.^6^ However, no apparent abnormalities were detected in nSMase1/*SMPD2* knockout mice, and no apparent abnormalities occurred in sphingomyelin metabolism.^17^ Human nSMase1 acted as a lyso-PAF phospholipase C when overexpressed in human kidney HEK293 cells but had no effect on sphingomyelin metabolism.^36^ Overexpression of nSMase1 in human Jurkat cells had no effect on cluster of differentiation 95 (CD95)/Fas receptor-induced ceramide production or apoptosis.^37^ Overexpression of nSMase1 in the Michigan Cancer Foundation-7 breast cancer cell line (MCF-7) cells had only a small effect on sphingomyelin and ceramide levels as compared with overexpression of nSMase2.^35^ Taken together, these previous and the current findings suggest that the roles of nSMase1 might be limited to the sphingomyelin signaling pathway only in cells with active JNK signaling under certain stress conditions. Nevertheless, further studies are required to determine the significance of the effects of JNK-mediated phosphorylation of Ser-270 of nSMase1 on stress-induced ceramide generation and apoptosis in other cell types and animal models, including *SMPD2* knockout mice and zebrafish. In addition, whether different stimuli that induce apoptosis independently of ceramide generation might be mediated via JNK signaling should be investigated.

Human, mouse, and zebrafish nSMase1 localizes to the ER and Golgi apparatus.^13, 14, 16^ In addition, Mizutani *et al.*^33^ reported that nSMase1 localizes to the nuclear matrix in rat cells. Our results indicate that phosphorylated nSMase1 is found in both the microsomal and nuclear fractions under stress condition and upon Fas stimulation. This suggests that nSMase1 might mediate ceramide generation, apoptosis, and other related cellular functions via JNK signaling under stress conditions in the microsomal and nuclear compartments. The localization of the phosphorylated activated form of nSMase1 in the ER and Golgi apparatus is consistent with the cellular functions of the enzyme in ceramide generation for vesicle-membrane fusion and exocytosis.^38^ nSMase1 has been reported to induce aggregation and fusion by ceramide release^39^ and to disturb the lipid bilayer structure in favor of a non-lamellar and micellar phase.^40^ Thus nSMase1 activation and ceramide generation may be induced by local vesicle formation and fusion under stress conditions. Additionally, a possibility exists that ceramide generated in such intracellular compartments during the apoptotic process might activate the caspase cascade under stress conditions. Cathepsin D,^41^ cytosolic phospholipase A2,^42^ and ceramide transfer protein,^43^ have been identified as molecular targets of ceramide action. The ceramide-binding protein that enhances caspase-3 activation and induces apoptosis via nSMase1 activity remains to be established. Through immunocytochemical staining, weak signals were detected in the nucleus under non-stress conditions (Supplementary Figure S16). Phosphorylated nSMase1 was also observed in the nuclear fraction under non-stress conditions. Ceramide may have an important role in the biosynthesis of nuclear membrane via sphingolipid metabolism.

The present findings demonstrate that phosphorylation of nSMase1 on Ser-270 is the crucial step in stress-induced ceramide generation and apoptosis. In addition to this, other studies show that apoptosis can be induced in a manner that is independent of ceramide generation. For example, mitomycin C and sodium azide can activate caspases via a ceramide-independent pathway and are not inhibited by cytokine response modifier A in MCF-7 cells.^44^ Palmitate is able to induce apoptosis independently of ceramide generation in cell lines, such as, rat hepatoma H4IIE cells,^45^ LY-B cells (Chinese hamster ovary (CHO) cells that lacked the LCB1 protein),^46^ and a mutant CHO cell line that lacks palmitoyltransferase activity to catalyze the rate-limiting step of *de novo* ceramide synthesis.^47^ When ceramide synthetase was inhibited by fumonisin B1 in LY-B cells, *de novo* ceramide synthesis was not essential for palmitate-induced apoptosis.^48^ In palmitate-treated cardiac myocytes, cytochrome *c* release and caspase-3 activation preceded ceramide accumulation.^49^ Palmitate-mediated production of reactive oxygen species may cause significant cellular dysfunction that contributes to the pathogenesis of these diseases prior to cell death.^48^

Cytokines, including Fas ligand and TNF-*α*, mediate ceramide generation. In response to TNF-*α*-mediated inflammation, nSMase2 and nSMase3 were reported to be downstream of the factor associated with nSMase activity.^12^ Three known signaling pathways can result in apoptosis upon Fas stimulation: (1) caspase-8-mediated caspase-3 and -7 activation,^50^ (2) caspase-8-mediated proteolytic processing of BH3 interacting-domain death agonist (Bid) into truncated Bid, which transmits a signal to the mitochondria to the release cytochrome *c*^51, 52^ and (3) death domain-associated protein-mediated apoptosis signal-regulating kinase 1 activation, followed by sequential phosphorylation/activation of MKK4/7, JNK, and B-cell lymphoma 2 interacting mediator of cell death.^53, 54, 55, 56, 57^ Our present study reveals that an anti-Fas antibody can induce caspase-3 activation at 2 h, in addition to phosphorylation of both JNK1/2 and nSMase1 at 3 h after treatment, in Jurkat T cells. Previously, Watts *et al.*^58^ showed that Fas-induced apoptosis of T cells occurred independently of ceramide generation. No changes in intracellular ceramide levels were observed up to 2 h poststimulation of Jurkat T cells treated with an anti-Fas IgM, although this treatment did induce apoptosis as assessed based on detection of hypodiploid DNA content by flow cytometry. However, a possibility exists that ceramide generation occurs at the late stages after Fas stimulation, which has not been fully addressed by Watts *et al.*^58^ Caricchio *et al.*^59^ studied ceramide-induced apoptosis in Jurkat cell clones selected for resistance to membrane-bound FasL-induced death. They demonstrated that ceramide was a second messenger for the Fas/FasL pathway and that serum withdrawal, through the production of ceramide, shared a common step with the Fas-mediated apoptotic pathway. Despite the JNK activation, FasL expression was not upregulated after apoptosis induced by both exogenous and endogenous ceramide, suggesting that JNK activation was directly upstream of the final steps of apoptosis. Therefore, in contrast to stress induced apoptosis, in which ceramide generation induces caspase-3 activation, Fas-induced apoptosis may involve ceramide generation via phosphorylated activation of JNK signaling and subsequent nSMase1 activation following caspase-8-mediated caspase-3 activation.

## Materials and Methods

### Materials

C_6_-7-Nitro-2-1,3-benzoxadiazol-4-yl (C_6_-NBD) sphingomyelin was obtained from Matreya (Pleasant Gap, PA, USA). Anthra[1,9-cd]pyrazol-6(2H)-one (SP600125, a JNK inhibitor), the p3 × FLAGCMV-14 expression vector, mouse anti-nSMase1/*SMPD2* monoclonal (2C9), mouse anti-FLAG M2 monoclonal, mouse anti-actin monoclonal, and mouse anti-tubulin monoclonal antibodies were purchased from Sigma-Aldrich (St. Louis, MO, USA). Cell culture reagents, mouse anti-V5 monoclonal antibody, pcDNA™6/V5-His A expression vector, and fetal bovine serum (FBS) were obtained from Invitrogen (Carlsbad, CA, USA). PrimeSTAR HS DNA polymerase and PrimeSTAR Mutagenesis Basal Kit were purchased from Takara Biomedical (Otsu, Shiga, Japan). Mouse recombinant JNK1 protein, and rabbit anti-pan-cadherin polyclonal, rabbit anti-SAPK/JNK polyclonal, rabbit anti-phospho-SAPK/JNK (Thr183/Tyr185, 81E11) monoclonal, mouse anti-phospho-SAPK/JNK (Thr183/Tyr185, G9) monoclonal, rabbit anti-SEK1/MKK4 (5C10) monoclonal, rabbit anti-phospho-SEK1/MKK4 (Ser257/Thr261) monoclonal, rabbit anti-MKK7 polyclonal, rabbit anti-phosopho-MKK7 (Ser271/Thr275) monoclonal, anti-phospho-c-Jun (Ser73) monoclonal, and rabbit anti-histone H2A.Z polyclonal antibodies were purchased from Cell Signaling Technology (Beverly, MA, USA). Goat anti-aldolase A (N-15) polyclonal antibody was obtained from Santa Cruz Biotechnology (Santa Cruz, CA, USA). Mouse anti-Fas (CD95) monoclonal antibody was purchased from Roche (Penzberg, Germany). [*γ*-^32^P]-ATP (370 MBq mM/ml), Hybond-P polyvinylidene fluoride (PVDF) membrane, protein G-Sepharose, and secondary antibodies were acquired from GE Healthcare (Piscataway, NJ, USA). All other chemicals were obtained from Wako Pure Chemicals (Osaka, Japan).

### Cell culture

The zebrafish cell line ZE, derived from embryos, was cultured in Leibovitz's L-15 medium (Invitrogen), supplemented with 2% heat-inactivated FBS (Invitrogen) at 25 °C.^16^ Jurkat T cells were obtained from RIKEN (Saitama, Japan),^28^ and were maintained in RPMI-1640 medium containing heat-inactivated 10% FBS at 37 °C in a humidified incubator with 5% CO_2_. Cells in the exponential growth phase were resuspended in 2% FBS-containing media at a concentration of 3 × 10^5^ cells/ml and then treated.

### Phospho-nSMase1 peptide and antibody production

The phospho-nSMase1 antibody was custom synthesized by JBioS (Saitama, Japan). Briefly, two peptides were synthesized: peptide 1 (DKPFP{phosphoSer270}DHEALMAD) and peptide 2 (DKPFPSDHEALMAD). The integrity of the peptides was verified by HPLC and mass spectroscopy. The peptides were conjugated to keyhole limpet hemocyanin through an added cysteine residue in the C-terminal region. Two rabbits were used for the immunizations. The fourth bleed serum was double-affinity-purified by consecutive elution through two columns using peptides 1 and 2. An ELISA was performed on the crude, flow-through, and eluent from both columns. The specificity of the antibody was demonstrated using peptide 1, which competed for the phospho-nSMase1 epitope in western blotting experiments.

### Stress treatments

ZE cells were plated at 5 × 10^5^ cells/ml in preheated medium in culture dishes, heat-shocked in an incubator at 38 °C, and allowed to recover at 25 °C.^16^ The cells were then stained with DAPI, and at least 200 cells were counted under a light microscope (IX71, Olympus, Tokyo, Japan).^16, 34^ Cells exhibiting nuclear condensation and fragmentation were judged to be apoptotic. For human Jurkat T cells, 3 × 10^5^ cells/ml were incubated at 37 °C in 4 ml of sterilized tap water in a 6-cm cell culture dish. For the heat-shock treatment, dishes containing cells were sealed with Parafilm and placed in water baths at 37, 43, or 43.5 °C for 1 h. For UV treatment, the cells were placed in culture dishes without covers and irradiated at 254 nm in a UV cross-linker (FS-800; Funakoshi, Tokyo, Japan). For H_2_O_2_ treatments, cells were treated with 0, 0.5, 1, or 2 mM H_2_O_2_. For Fas antibody treatments, 0, 25, 50, or 100 ng/ml anti-Fas antibody was added to cells. The human cells were then allowed to recover at 37 °C. For western blotting, nSMase and caspase-3 assays, the measurement of ceramide content, and apoptotic DAPI staining, the stress-treated cells were collected 0–12 h posttreatment.

### Caspase-3 assay

Caspase-3 activity was measured using the synthetic substrate acetyl-DEVD-4-methyl-coumaryl-7-amide as described previously.^43^ The release of 7-amino-4-methyl-coumarin (AMC) was measured using a fluorescent spectrophotometer (Molecular Devices, Sunnyvale, CA, USA) with an excitation at 360 nm and an emission at 450 nm. Protein concentrations were determined using a protein assay kit (Bio-Rad, Hercules, CA, USA). One unit of enzyme activity was defined as the release of 1 nmol AMC per hour at 37 °C.

### Western blotting

Proteins extracted from the immunoprecipitants of whole cells, the microsomal fraction, the cytosolic fraction, or nuclear fraction were resolved on 10 or 15% SDS-polyacrylamide gels and electroblotted onto PVDF membranes as described by Yabu *et al.*^16, 60^ Anti-FLAG monoclonal, anti-V5 monoclonal, anti-nSMase1 polyclonal,^16^ anti-nSMase1/SMPD2 monoclonal, anti-phospho-nSMase1 polyclonal, anti-actin monoclonal, anti-pan-cadherin polyclonal, anti-aldolase polyclonal, anti-tubulin monoclonal, anti-JNK polyclonal, rabbit anti-phospho-JNK (Thr183/Tyr185) monoclonal, mouse anti-phospho-JNK (Thr183/Tyr185) monoclonal, anti-phospho-MKK4 (Ser257/Thr261) polyclonal, anti-phospho-MKK7 (Ser271/Thr275) polyclonal, anti-phospho-c-Jun (Ser73) polyclonal, anti-SEK1/MKK4 (5C10) monoclonal, anti-MKK7 polyclonal, and anti-histone H2A.Z polyclonal primary antibodies were used. Following reactions with the appropriate secondary antibodies, the resulting signals were detected by chemiluminescence using a Western Lightning ECL Pro kit (PerkinElmer Inc., Waltham, MA, USA) according to the manufacturer's instructions.

### Subcellular fractionation

Subcellular fractionation was performed as described previously.^16, 60, 61^ The purity of all fractions in each experiment was determined by western blotting for aldolase (cytosol) and cadherin (microsomes).

### Nuclear fractionation

Nuclei fractionation was performed as described previously with slight modifications.^28^ Briefly, the cells were lysed by passing through a 27-gauge needle in a lysis buffer containing 10 mM Tris–HCl (pH 7.5), 30 mM KCl, 5 mM MgCl_2_, 1 × protease inhibitor mix (Roche), and 1 × phosphatase inhibitor (Roche). After incubation on ice for 15 min, the lysed cells were mixed with 1 M sucrose. A nuclear pellet was obtained after centrifugation at 1600 × *g* for 10 min at 4 °C, resuspended in lysis buffer, and recentrifuged at 1600 × *g* for 10 min at 4 °C. The fractionation purity was confirmed by western blotting for the nuclear marker histone H2A.

### Immunofluorescence microscopy

Stressed Jurkat T cells were fixed with 4% paraformaldehyde in phosphate-buffered saline (PBS) for 15 min, rinsed with PBS, and permeabilized with PBS containing 0.1% Triton X-100 for 3 min at room temperature. After incubation with PBS containing 2% bovine serum albumin for 1 h, the samples were cross-reacted with both 1.5 *μ*g/ml of anti-phospho nSMase1 rabbit polyclonal IgG and an antibody for a subcellular marker (1 *μ*g/ml of mouse anti-calnexin monoclonal IgG (AF18) or 10 *μ*g/ml of mouse anti-58-k Golgi protein monoclonal IgG (58K-9)) in blocking buffer at 4 °C, overnight; the antibodies were purchased from Abcam (Cambridge, UK). After washing three times for 15 min with PBS containing 0.1% Tween 20, the samples were cross reacted with Alexa Fluor 488-labeled goat anti-rabbit IgG and Alexa Fluor 594-labeled goat anti-mouse IgG secondary antibodies for 3 h (1 : 250; both from Invitrogen). After three washes for 15 min with PBS containing 0.1% Tween 20, the cells were then counterstained with TOTO-3 (Invitrogen) and viewed under a laser-scanning confocal microscope (LSM 510, Carl Zeiss, Wetzlar, Germany).

### SMase assays

Enzymatic assays to assess sphingomyelinase activity were performed using C_6_-NBD-sphingomyelin. The assays were performed as described previously.^16^ One unit of enzyme activity was defined as the release of 100 pmol C_6_-NBD-ceramide per hour at 37 °C.

### Ceramide measurement

Lipids were extracted from whole cells as described by Bligh and Dyer,^62^ and the ceramide contents were measured using *Escherichia coli* diacylglycerol kinase as described previously.^63^ The solvent used to separate ceramide 1-phosphate phosphate was chloroform/acetone/methanol/acetic acid/water (10 : 4 : 3 : 2 : 1, v/v). The ceramide content was measured using a STORM 860 analyzer (GE Healthcare).

### Construction of the expression vector

JNK1 fusion constructs, K55R substitution mutants, and V5 tag-containing mutants were created using the PrimeSTAR Mutagenesis Basal Kit (Takara Biomedical) with zebrafish JNK1 cDNA and pcDNA6/V5-His A expression vector (Invitrogen), or derivatives, as the templates and the appropriate combinations of the forward and reverse oligonucleotide primers. An nSMase1-FLAG tag fusion construct and an S270A or S270E substitution mutant were generated using the PrimeSTAR Mutagenesis Basal Kit (Takara Biomedical) with zebrafish nSMase1 cDNA,^16^ p3 × FLAG-CMV-14 expression vector (Sigma-Aldrich), and pET-16b vector for the recombinant protein. The fidelity of the nSMase1 and JNK1 mutants was confirmed by sequencing. His-tagged recombinant proteins containing nSMase1 and various mutants were purified as described previously.^16^

### Transient transfection of DNA into cultured cells

Zebrafish ZE cells were cultured at a density of 1 × 10^6^ cells in 60-mm dishes in 4 ml of Leibovitz's L-15 medium supplemented with 2% FBS. At 90% confluence, cells were transiently transfected with three different concentrations (1, 2, or 3 *μ*g) of nSMase1 constructs (mock, wild-type, S270A mutant, and S270E mutant) or vector DNA using FuGENE 6 transfection reagent (Roche) following the manufacturer's instructions.

Jurkat T cells were cultured at a density of 2 × 10^6^ cells in 60-mm dishes in 4 ml of RPMI-1640 medium supplemented with 2% FBS. The cells were transfected with the nSMase1 mock, wild-type, S270A mutant, and S270E mutant constructs using *TransIT* Jurkat Transfection Reagent (Mirus Bio LLC, Madison, WI, USA). Briefly, 1, 2.5, or 5 *μ*g of vector DNA was added to 10 *μ*l *TransIT* Jurkat Reagent in 200 *μ*l of serum-free RPMI-1640 medium, added to cells, and then incubated for 24 h at 37 °C. nSMase assays, the measurement of ceramide content, caspase-3 assays, and apoptotic DAPI analysis were then performed. Cell viability was determined using the Trypan Blue dye exclusion method.^16^

### Generation of zebrafish cell stable transfectants

ZE cells were cultured in Leibovitz's L-15 medium supplemented with 2% FBS. To obtain stable transfectants, ZE cells were transfected with 4 *μ*g of either JNK1 wild-type, JNK1-K55R mutant (DN), nSMase1 wild-type,^16^ or nSMase1-S270A mutant (substitution of serine-270 to alanine) constructs using FuGENE 6 Transfection Reagent (Roche) following the manufacturer's instructions. Transfected cells were selected with 0.8-mg/ml geneticin (Nakalai Tesque, Kyoto, Japan). Cell lines that overexpressed the JNK1 wild-type, JNK1-K55R mutant, nSMase1 wild-type,^16^ and nSMase1-S270A mutant stably and at high levels were established.

### Immunoprecipitation

After stress exposures or anti-Fas antibody treatment, the cells were washed twice with ice-cold PBS buffer, and lysed for 1 h at 4 °C in 1 ml of 20 mM Tris–HCl (pH 7.5) containing 1% Nonidet P-40, 1% Triton X-100, 150 mM NaCl, 5 mM MgCl_2_, 1 mM EDTA, 1 mM EGTA, 10 mM sodium fluoride, 1 mM sodium orthovanadate, and 1 × proteinase inhibitor cocktail. Cellular debris was pelleted by centrifugation at 13 000 × *g* for 15 min at 4 °C. The resulting cell lysates were first incubated with anti-phospho or total JNK antibodies, followed by incubation with protein G Sepharose (GE Healthcare). The lysates were then sedimented and washed three times with 20 mM Tris–HCl (pH 7.5) containing 0.1% Triton X-100, 150 mM NaCl, 5 mM MgCl_2_, 1 mM EDTA, 1 mM EGTA, 10 mM sodium fluoride, 1 mM sodium orthovanadate, and 1 × proteinase inhibitor cocktail.

### Recombinant protein production

The purification of nSMase1 enzyme was performed as reported previously.^16^ The three nSMase1-recombinant proteins (nSMase1-wild-type, nSMase1-S270A mutant, and nSMase1-S270E mutant) were purified using two chromatography steps: sequential His-tag affinity purification, and gel filtration chromatography. The three recombinant proteins were used in *in vitro* kinase and nSMase enzyme assays.

### *In vitro* kinase assay

*In vitro* phosphorylation reactions were performed as follows: 100 ng of purified nSMase1 wild-type or nSMase1-S270A mutant enzyme were incubated with or without 2 ng of mouse JNK1 recombinant protein (Cell Signaling Technology) for 30 min at 30 °C in kinase buffer (20 mM HEPES–NaOH (pH 7.4), 10 mM MgCl_2_, 100 mM NaCl, 0.05% Triton X-100, 0.1 mM DTT, 0.25 mM ATP, and 18.5 MBq of [*γ*-^32^P]-ATP) in the presence or absence of 10 *μ*M SP600125. The mixture of JNK-post reaction was used in an nSMase assay. In addition, for western blotting, the reactions were terminated by the addition of 3 × SDS sample buffer (187.5 mM Tris–HCl (pH 6.8), 7.5% SDS, 30% glycerol, 2.14 M *β*-mercaptoethanol, and 0.006% bromophenol blue), and the samples were boiled for 5 min. Phosphorylated proteins were resolved by SDS-PAGE on 10% SDS-polyacrylamide gels and were then transferred onto PVDF membranes. nSMase1 phosphorylation was then detected by autoradiography.

### RNAi

To downregulate endogenous proteins in human Jurkat T cells, several RNAi oligonucleotides (Stealth RNAi, Invitrogen) were used: *MAPK8*, MAPK8HSS108549; *MAPK9*, MAPK9HSS108550; *SMPD2*, SMPD2HSS186000; *MAP2K4*, MAP2K4HSS184677; and *MAP2K7*, MAP2K4HSS140876. Nonspecific RNAi with medium GC content was used as a negative control (Stealth RNAi Negative Control Medium GC Duplex (45–2001)). For transfections, 5 × 10^6^ cells were transfected with 150 pmol of an equimolar mixture of the constructs using Xfect siRNA Transfection Reagent (Clontech, Mountain View, CA, USA) following the manufacturer's instructions. The knockdown of *MAPK8* and/or *MAPK9* was evident at 48 h. The western blot in Supplementary Figure S9 shows the knockdown 48 h after transfection. The efficiency of *SMPD2* knockdown was confirmed 24 h after transfection by western blotting (Figure 7a). The *MAP2K4* and/or *MAP2K7* knockdown was evident at 24 h. The western blot in Supplementary Figure S11a shows the knockdown 24 h after transfection.

### Statistical analysis

All data are presented as means±S.D.s. Differences among groups were analyzed using one-way analysis of variance followed by Bonferroni's *post hoc* test. Comparisons between the two experimental groups were performed using two-tailed *t-*tests.

## Abbreviations

AMC: 7-amino-4-methyl-coumarin
Bid: BH3 interacting-domain death agonist
C_6_-NBD: C_6_-7-nitro-2-1,3-benzoxadiazol-4-yl
CD95: cluster of differentiation 95
CHO: Chinese hamster ovary
DAPI: 4′,6-diamidino-2-phyenylidole
DN: dominant-negative
ER: endoplasmic reticulum
FBS: fetal bovine serum
IL-1*β*: interleukin-1*β*
JNK: c-Jun N-terminal kinase
LY-B cells: CHO cells that lacked the LCB1 protein
lyso-PAF: lyso-platelet-activating factor
MAPK: mitogen-activated protein kinase
MCF-7: Michigan Cancer Foundation-7 breast cancer cell line
MKK: MAPK kinase
nSMase: neutral sphingomyelinase
p38: p38 mitogen-activated protein kinases
PVDF: polyvinylidene fluoride
SAPK: stress-activated protein kinase
SEK: SAPK/ERK kinase
*SMPD2*: sphingomyelin phosphodiesterase 2
TNF: tumor necrosis factor
UV: ultraviolet
ZE cells: zebrafish embryonic cultured cells
z-VAD-fmk: carboxybenzyl-VAD-fluoromethyl ketone

## Notes

A patent application was submitted for phosphorylated nSMase1 and its specific antibody (No. 2012–272595, MY, SI, TY, KT). The remaining authors declare no conflict of interest.
