# Supplementary material for: Stress-induced ceramide generation and apoptosis via the phosphorylation and activation of nSMase1 by JNK signaling
Source: Cell Death Differ. 2014 Aug 29;22(2):258–73. doi: 10.1038/cdd.2014.128 (PMC4291487; doi:10.1038/cdd.2014.128)
Supplement: Supplementary Information [file cdd2014128x1.ppt]

## Slide 1
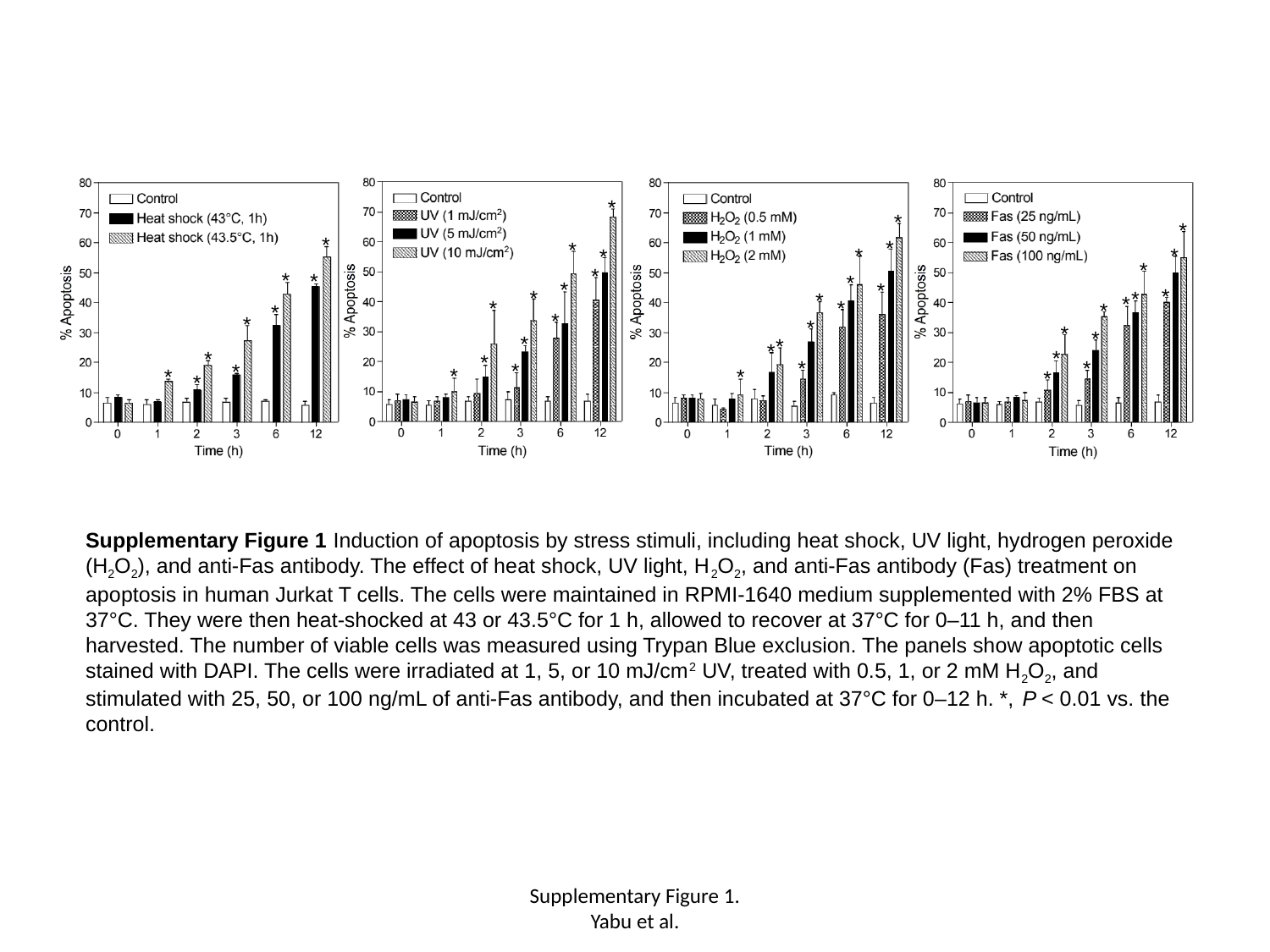

*
*
*
*
*
*
*
*
*
*
*
*
*
*
*
*
*
*
*
*
*
*
*
*
*
*
*
*
*
*
*
*
*
*
*
*
*
*
*
*
*
*
*
*
*
Supplementary Figure 1 Induction of apoptosis by stress stimuli, including heat shock, UV light, hydrogen peroxide (H2O2), and anti-Fas antibody. The effect of heat shock, UV light, H2O2, and anti-Fas antibody (Fas) treatment on apoptosis in human Jurkat T cells. The cells were maintained in RPMI-1640 medium supplemented with 2% FBS at 37°C. They were then heat-shocked at 43 or 43.5°C for 1 h, allowed to recover at 37°C for 0–11 h, and then harvested. The number of viable cells was measured using Trypan Blue exclusion. The panels show apoptotic cells stained with DAPI. The cells were irradiated at 1, 5, or 10 mJ/cm2 UV, treated with 0.5, 1, or 2 mM H2O2, and stimulated with 25, 50, or 100 ng/mL of anti-Fas antibody, and then incubated at 37°C for 0–12 h. *, P < 0.01 vs. the control.
Supplementary Figure 1.
Yabu et al.

## Slide 2
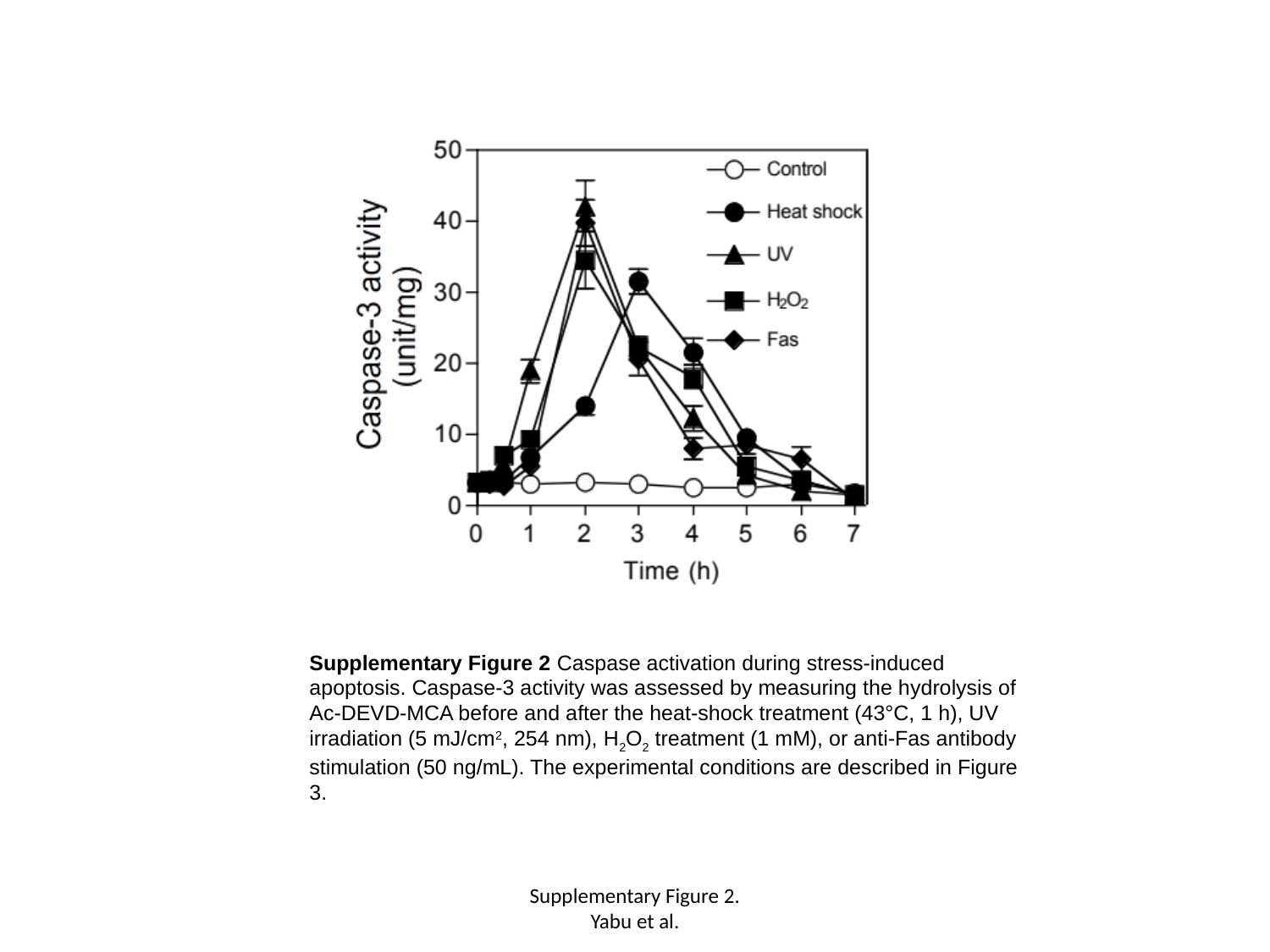

Supplementary Figure 2 Caspase activation during stress-induced apoptosis. Caspase-3 activity was assessed by measuring the hydrolysis of Ac-DEVD-MCA before and after the heat-shock treatment (43°C, 1 h), UV irradiation (5 mJ/cm2, 254 nm), H2O2 treatment (1 mM), or anti-Fas antibody stimulation (50 ng/mL). The experimental conditions are described in Figure 3.
Supplementary Figure 2.
Yabu et al.

## Slide 3
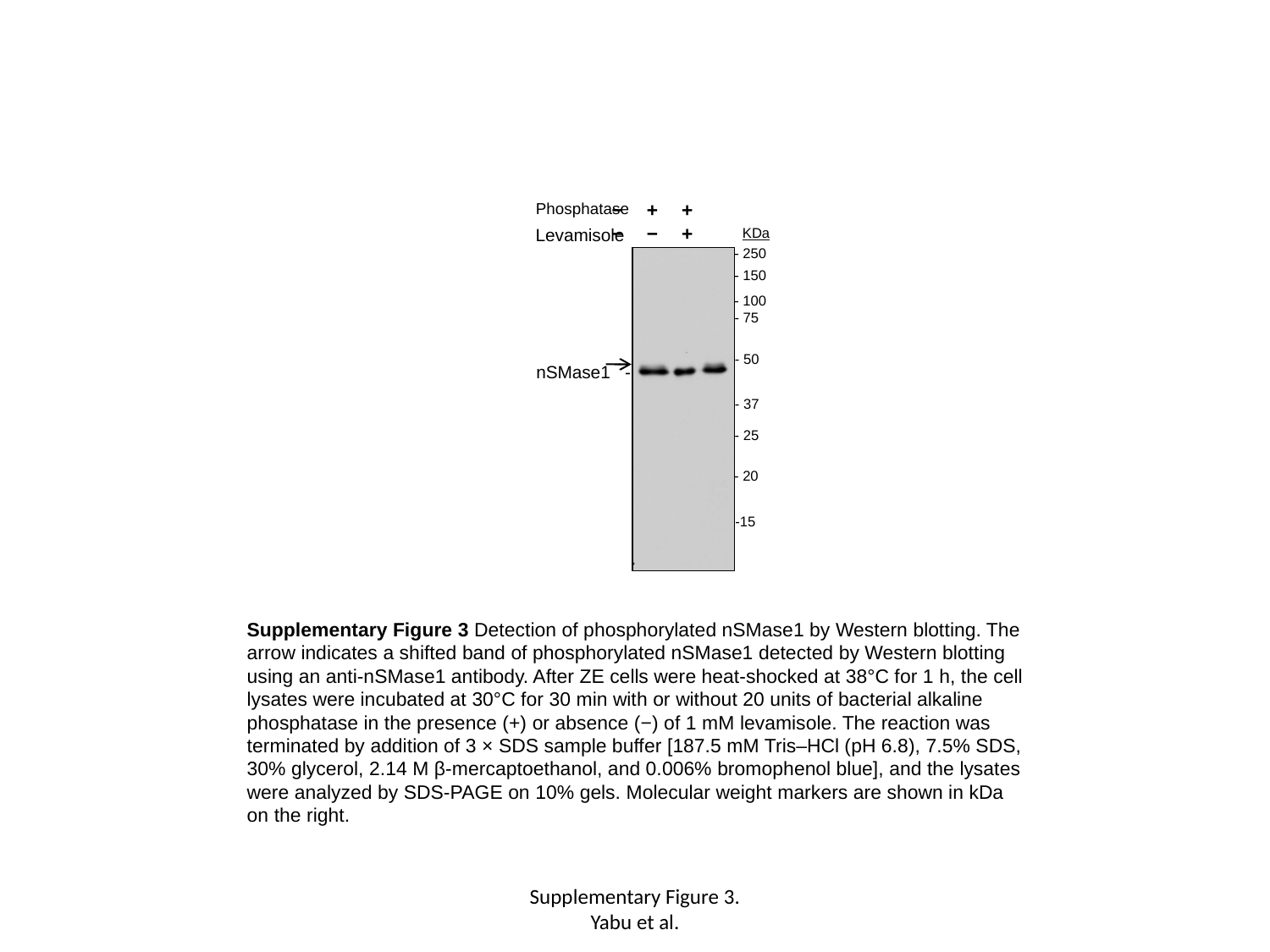

− + +
− − +
Phosphatase
Levamisole
KDa
- 250
- 150
- 100
- 75
- 50
nSMase1 -
- 37
- 25
- 20
-15
Supplementary Figure 3 Detection of phosphorylated nSMase1 by Western blotting. The arrow indicates a shifted band of phosphorylated nSMase1 detected by Western blotting using an anti-nSMase1 antibody. After ZE cells were heat-shocked at 38°C for 1 h, the cell lysates were incubated at 30°C for 30 min with or without 20 units of bacterial alkaline phosphatase in the presence (+) or absence (−) of 1 mM levamisole. The reaction was terminated by addition of 3 × SDS sample buffer [187.5 mM Tris–HCl (pH 6.8), 7.5% SDS, 30% glycerol, 2.14 M β-mercaptoethanol, and 0.006% bromophenol blue], and the lysates were analyzed by SDS-PAGE on 10% gels. Molecular weight markers are shown in kDa on the right.
Supplementary Figure 3.
Yabu et al.

## Slide 4
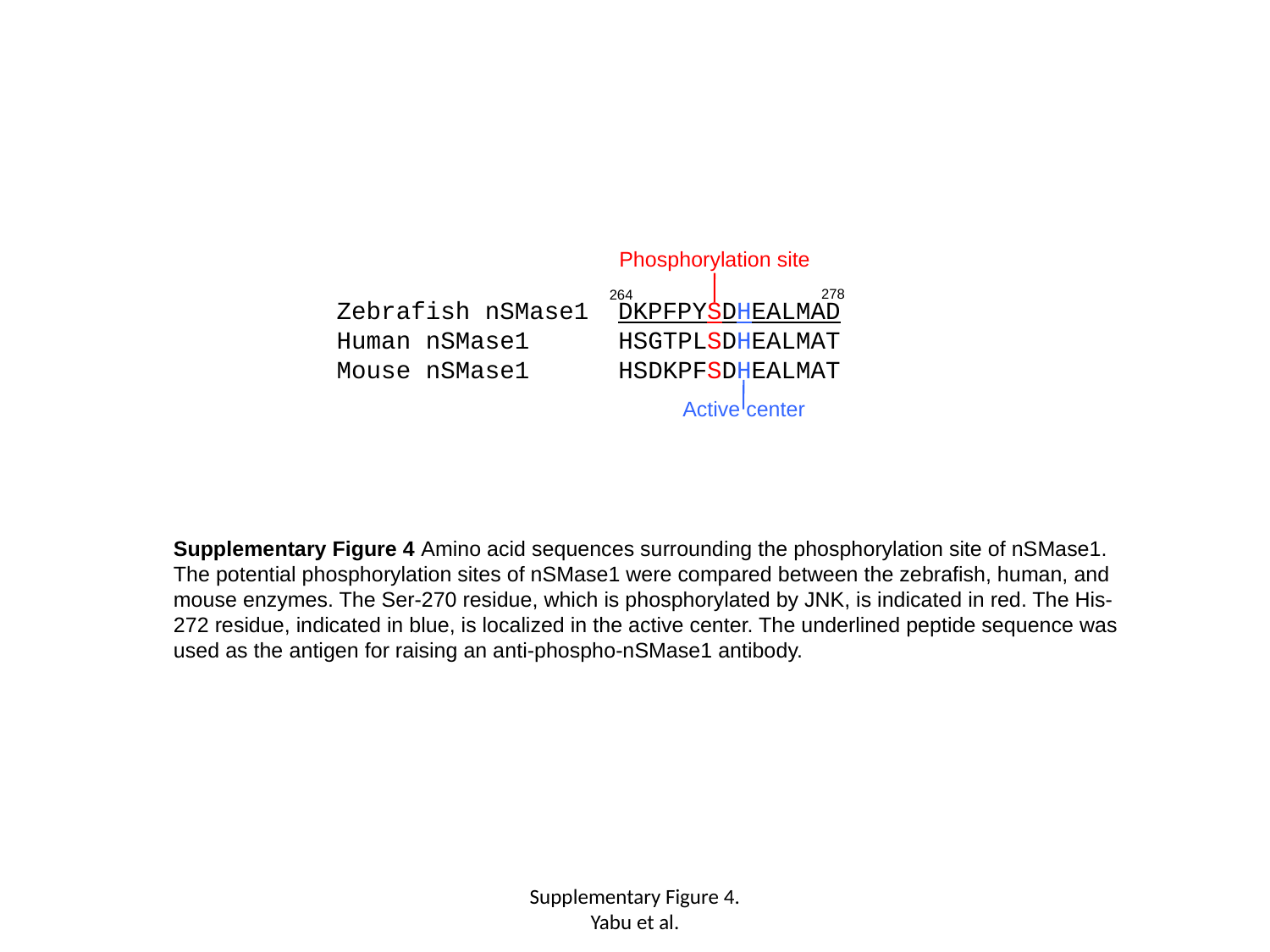

Phosphorylation site
Zebrafish nSMase1 DKPFPYSDHEALMAD
Human nSMase1 HSGTPLSDHEALMAT
Mouse nSMase1 HSDKPFSDHEALMAT
Active center
278
264
Supplementary Figure 4 Amino acid sequences surrounding the phosphorylation site of nSMase1. The potential phosphorylation sites of nSMase1 were compared between the zebrafish, human, and mouse enzymes. The Ser-270 residue, which is phosphorylated by JNK, is indicated in red. The His-272 residue, indicated in blue, is localized in the active center. The underlined peptide sequence was used as the antigen for raising an anti-phospho-nSMase1 antibody.
Supplementary Figure 4.
Yabu et al.

## Slide 5
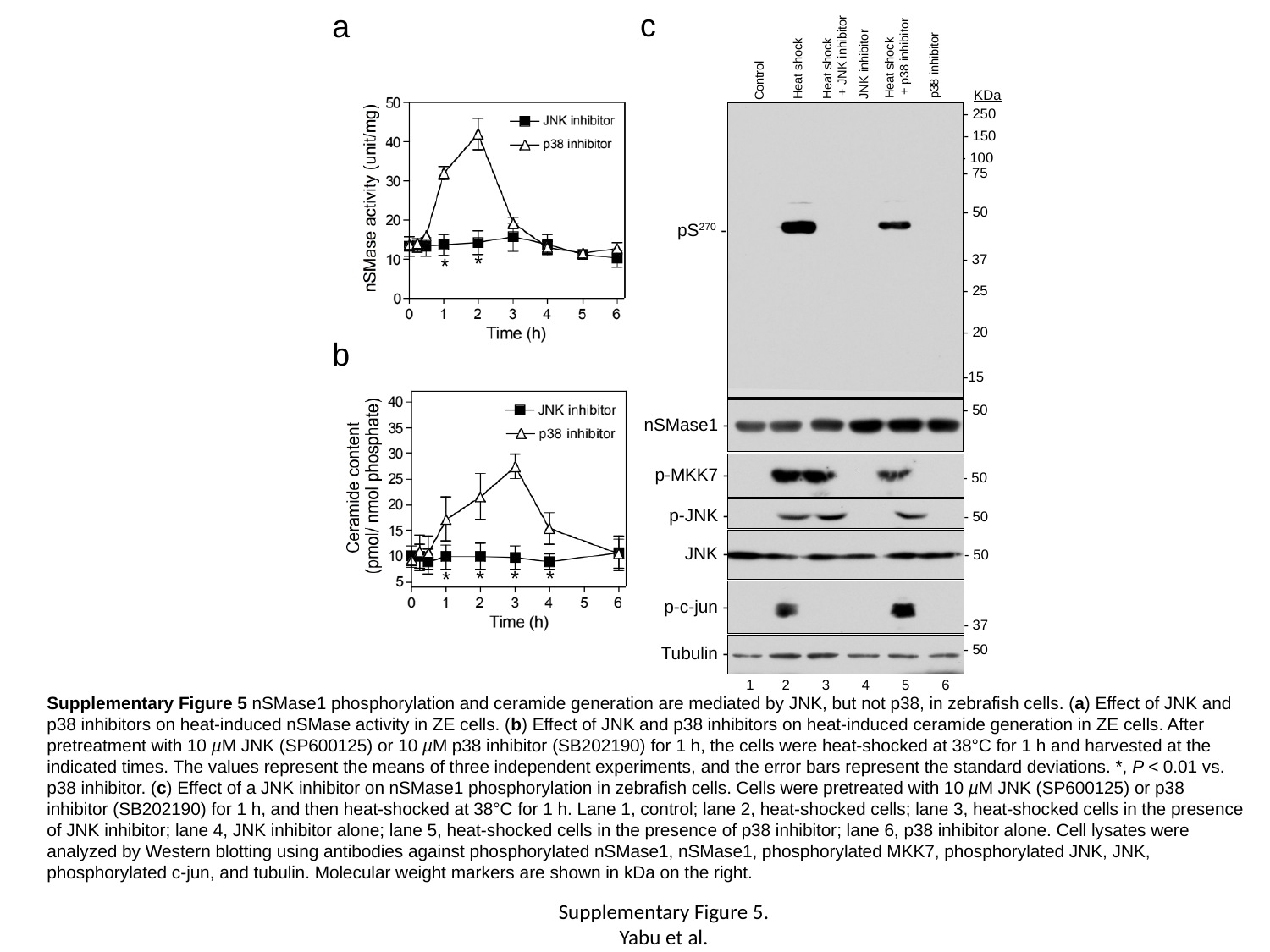

c
Heat shock
 + JNK inhibitor
Heat shock
 + p38 inhibitor
 JNK inhibitor
 p38 inhibitor
Heat shock
Control
KDa
- 250
- 150
- 100
- 75
- 50
- 37
- 25
- 20
-15
pS270 -
- 50
nSMase1 -
p-MKK7 -
- 50
p-JNK -
- 50
JNK -
- 50
p-c-jun -
- 37
- 50
Tubulin -
1 2 3 4 5 6
a
*
*
b
*
*
*
*
Supplementary Figure 5 nSMase1 phosphorylation and ceramide generation are mediated by JNK, but not p38, in zebrafish cells. (a) Effect of JNK and p38 inhibitors on heat-induced nSMase activity in ZE cells. (b) Effect of JNK and p38 inhibitors on heat-induced ceramide generation in ZE cells. After pretreatment with 10 µM JNK (SP600125) or 10 µM p38 inhibitor (SB202190) for 1 h, the cells were heat-shocked at 38°C for 1 h and harvested at the indicated times. The values represent the means of three independent experiments, and the error bars represent the standard deviations. *, P < 0.01 vs. p38 inhibitor. (c) Effect of a JNK inhibitor on nSMase1 phosphorylation in zebrafish cells. Cells were pretreated with 10 µM JNK (SP600125) or p38 inhibitor (SB202190) for 1 h, and then heat-shocked at 38°C for 1 h. Lane 1, control; lane 2, heat-shocked cells; lane 3, heat-shocked cells in the presence of JNK inhibitor; lane 4, JNK inhibitor alone; lane 5, heat-shocked cells in the presence of p38 inhibitor; lane 6, p38 inhibitor alone. Cell lysates were analyzed by Western blotting using antibodies against phosphorylated nSMase1, nSMase1, phosphorylated MKK7, phosphorylated JNK, JNK, phosphorylated c-jun, and tubulin. Molecular weight markers are shown in kDa on the right.
Supplementary Figure 5.
Yabu et al.

## Slide 6
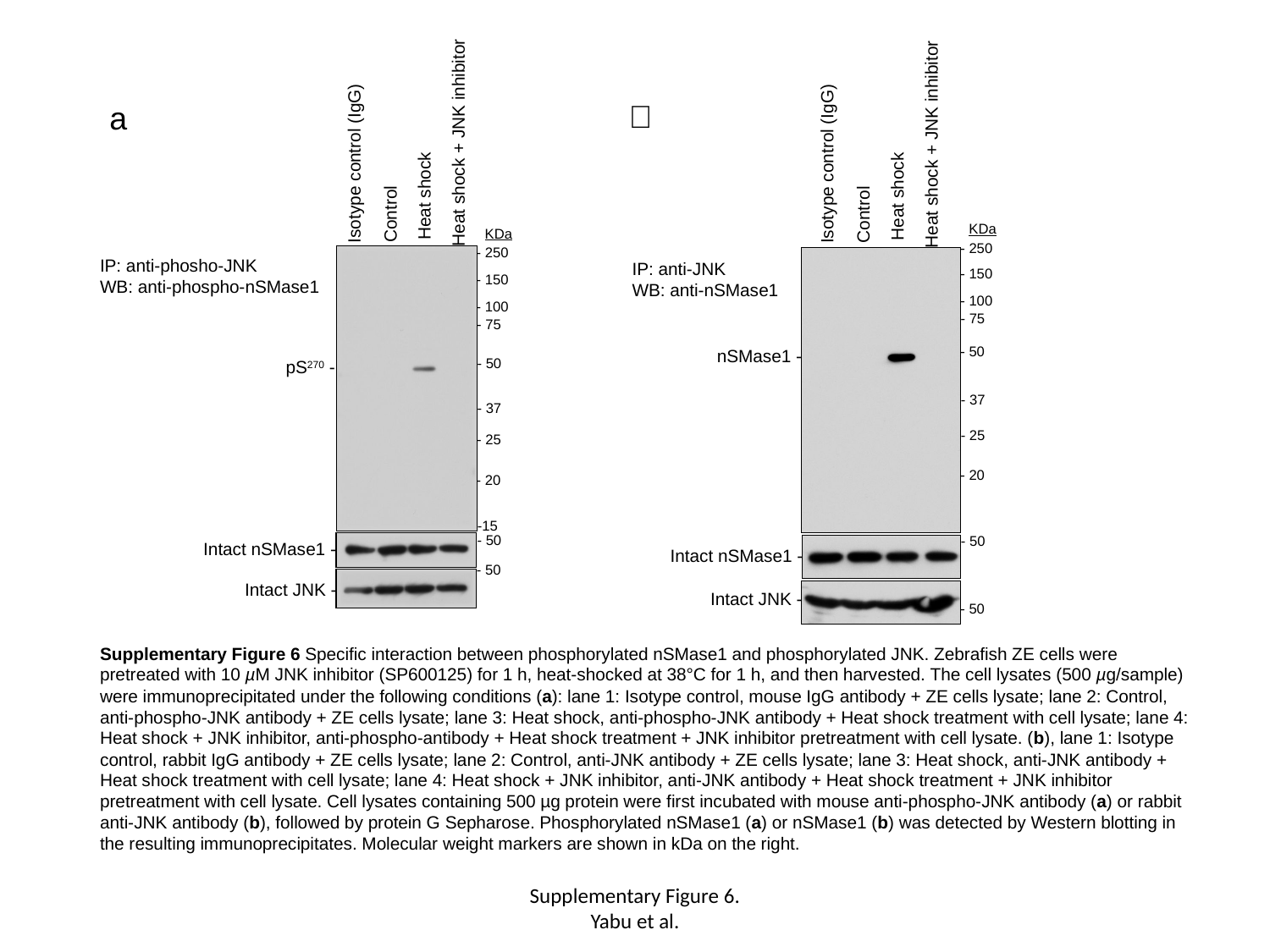

Heat shock + JNK inhibitor
Isotype control (IgG)
Heat shock
Control
KDa
- 250
IP: anti-phosho-JNK
WB: anti-phospho-nSMase1
- 150
- 100
- 75
- 50
pS270 -
- 37
- 25
- 20
-15
- 50
Intact nSMase1 -
- 50
Intact JNK -
a
Heat shock + JNK inhibitor
Isotype control (IgG)
Heat shock
Control
ｂ
KDa
- 250
- 150
- 100
- 75
- 50
- 37
- 25
- 20
- 50
- 50
IP: anti-JNK
WB: anti-nSMase1
nSMase1 -
Intact nSMase1 -
Intact JNK -
Supplementary Figure 6 Specific interaction between phosphorylated nSMase1 and phosphorylated JNK. Zebrafish ZE cells were pretreated with 10 µM JNK inhibitor (SP600125) for 1 h, heat-shocked at 38°C for 1 h, and then harvested. The cell lysates (500 µg/sample) were immunoprecipitated under the following conditions (a): lane 1: Isotype control, mouse IgG antibody + ZE cells lysate; lane 2: Control, anti-phospho-JNK antibody + ZE cells lysate; lane 3: Heat shock, anti-phospho-JNK antibody + Heat shock treatment with cell lysate; lane 4: Heat shock + JNK inhibitor, anti-phospho-antibody + Heat shock treatment + JNK inhibitor pretreatment with cell lysate. (b), lane 1: Isotype control, rabbit IgG antibody + ZE cells lysate; lane 2: Control, anti-JNK antibody + ZE cells lysate; lane 3: Heat shock, anti-JNK antibody + Heat shock treatment with cell lysate; lane 4: Heat shock + JNK inhibitor, anti-JNK antibody + Heat shock treatment + JNK inhibitor pretreatment with cell lysate. Cell lysates containing 500 µg protein were first incubated with mouse anti-phospho-JNK antibody (a) or rabbit anti-JNK antibody (b), followed by protein G Sepharose. Phosphorylated nSMase1 (a) or nSMase1 (b) was detected by Western blotting in the resulting immunoprecipitates. Molecular weight markers are shown in kDa on the right.
Supplementary Figure 6.
Yabu et al.

## Slide 7
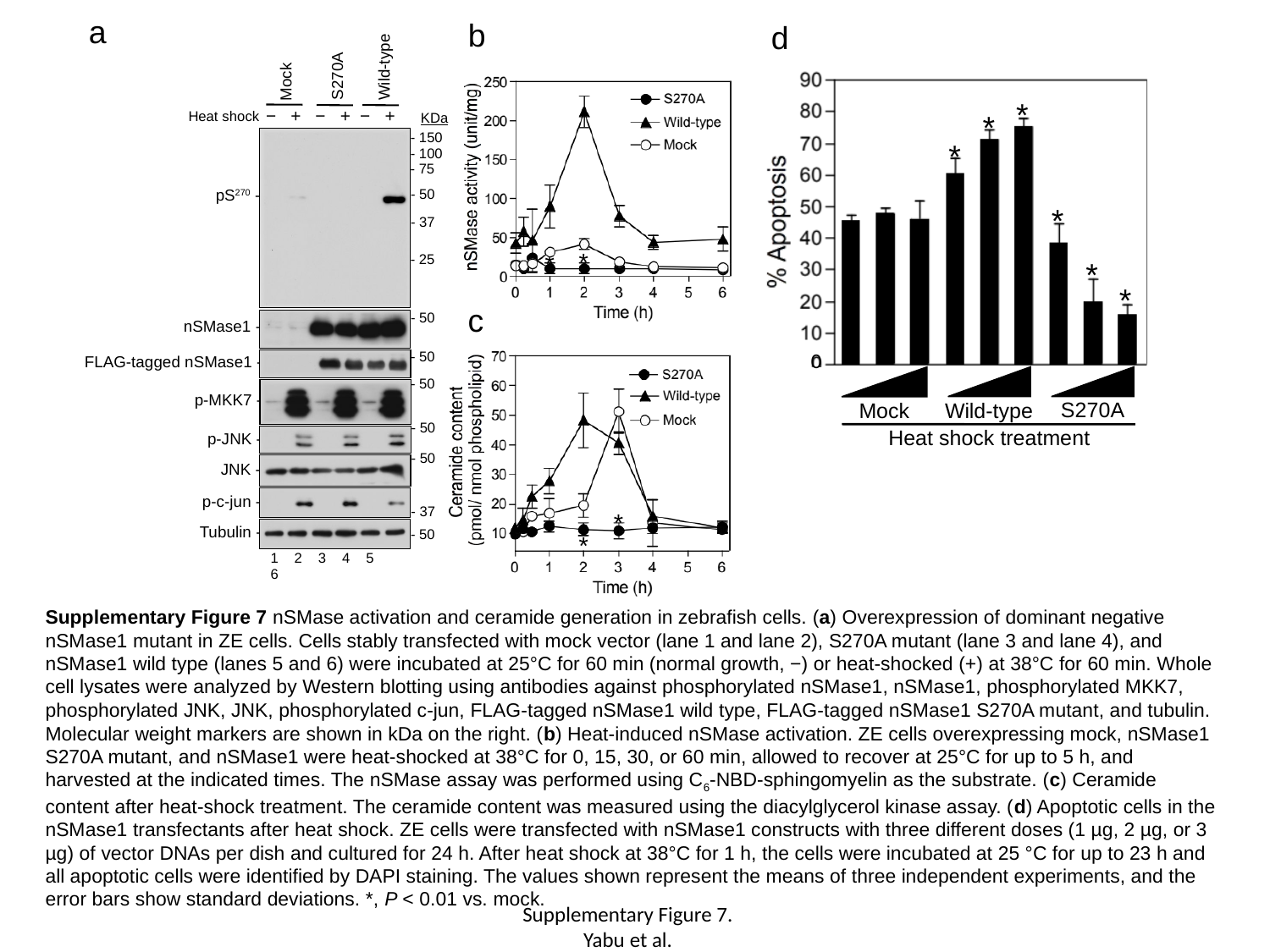

a
Wild-type
S270A
Mock
Heat shock
− + − + − +
KDa
- 150
pS270 -
- 100
- 75
- 50
- 37
- 25
- 50
nSMase1 -
- 50
FLAG-tagged nSMase1 -
- 50
p-MKK7 -
- 50
p-JNK -
- 50
JNK -
p-c-jun -
- 37
Tubulin -
1 2 3 4 5 6
- 50
b
*
*
c
*
*
d
*
*
*
*
*
*
0
Mock
Wild-type
S270A
Heat shock treatment
Supplementary Figure 7 nSMase activation and ceramide generation in zebrafish cells. (a) Overexpression of dominant negative nSMase1 mutant in ZE cells. Cells stably transfected with mock vector (lane 1 and lane 2), S270A mutant (lane 3 and lane 4), and nSMase1 wild type (lanes 5 and 6) were incubated at 25°C for 60 min (normal growth, −) or heat-shocked (+) at 38°C for 60 min. Whole cell lysates were analyzed by Western blotting using antibodies against phosphorylated nSMase1, nSMase1, phosphorylated MKK7, phosphorylated JNK, JNK, phosphorylated c-jun, FLAG-tagged nSMase1 wild type, FLAG-tagged nSMase1 S270A mutant, and tubulin. Molecular weight markers are shown in kDa on the right. (b) Heat-induced nSMase activation. ZE cells overexpressing mock, nSMase1 S270A mutant, and nSMase1 were heat-shocked at 38°C for 0, 15, 30, or 60 min, allowed to recover at 25°C for up to 5 h, and harvested at the indicated times. The nSMase assay was performed using C6-NBD-sphingomyelin as the substrate. (c) Ceramide content after heat-shock treatment. The ceramide content was measured using the diacylglycerol kinase assay. (d) Apoptotic cells in the nSMase1 transfectants after heat shock. ZE cells were transfected with nSMase1 constructs with three different doses (1 µg, 2 µg, or 3 µg) of vector DNAs per dish and cultured for 24 h. After heat shock at 38°C for 1 h, the cells were incubated at 25 °C for up to 23 h and all apoptotic cells were identified by DAPI staining. The values shown represent the means of three independent experiments, and the error bars show standard deviations. *, P < 0.01 vs. mock.
Supplementary Figure 7.
Yabu et al.

## Slide 8
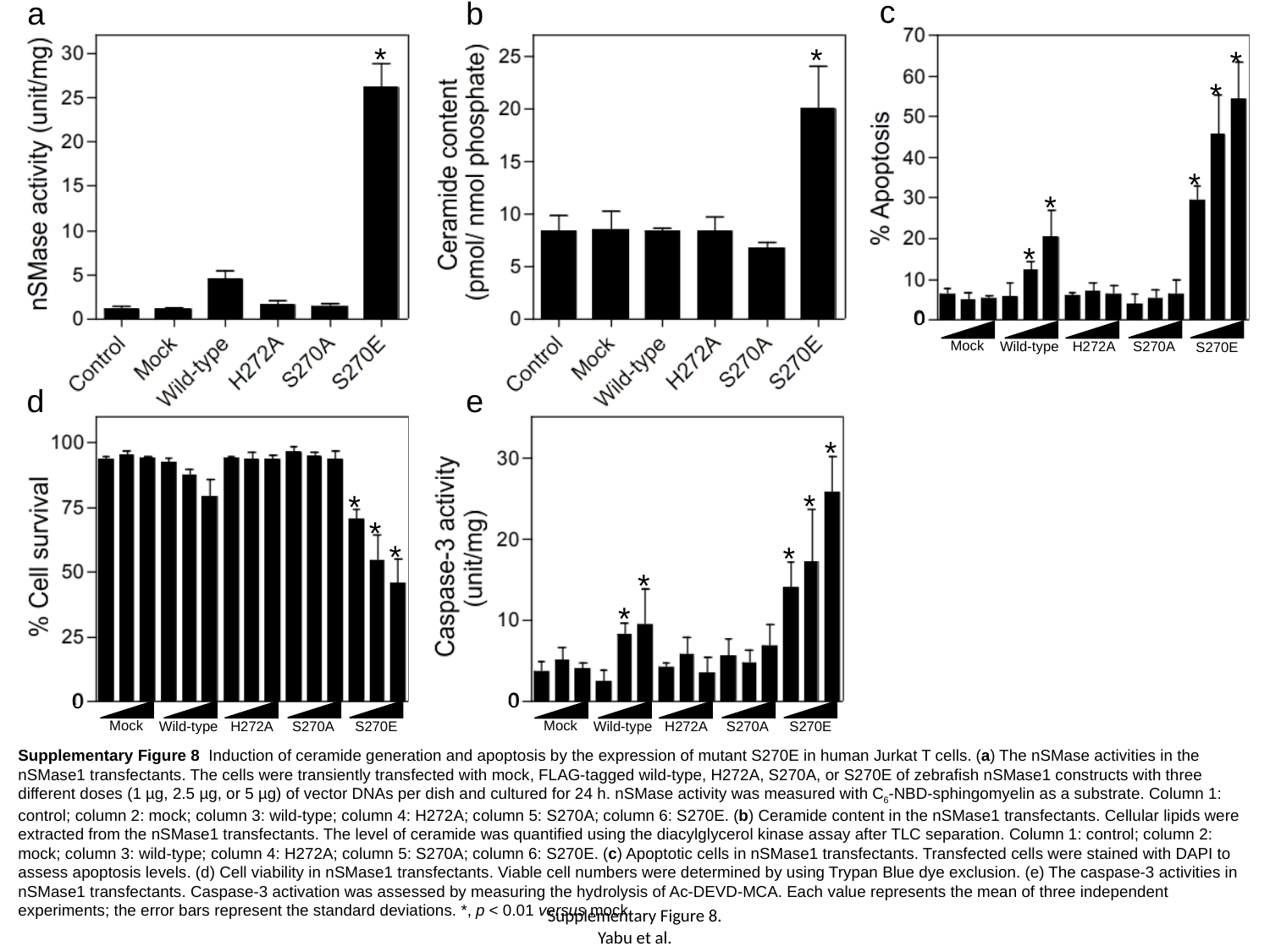

c
*
*
*
*
*
0
Mock
Wild-type
H272A
S270A
S270E
a
b
*
*
d
e
Mock
Wild-type
H272A
S270A
S270E
*
*
*
Mock
Wild-type
H272A
S270A
S270E
*
*
*
*
*
0
0
Supplementary Figure 8 Induction of ceramide generation and apoptosis by the expression of mutant S270E in human Jurkat T cells. (a) The nSMase activities in the nSMase1 transfectants. The cells were transiently transfected with mock, FLAG-tagged wild-type, H272A, S270A, or S270E of zebrafish nSMase1 constructs with three different doses (1 µg, 2.5 µg, or 5 µg) of vector DNAs per dish and cultured for 24 h. nSMase activity was measured with C6-NBD-sphingomyelin as a substrate. Column 1: control; column 2: mock; column 3: wild-type; column 4: H272A; column 5: S270A; column 6: S270E. (b) Ceramide content in the nSMase1 transfectants. Cellular lipids were extracted from the nSMase1 transfectants. The level of ceramide was quantified using the diacylglycerol kinase assay after TLC separation. Column 1: control; column 2: mock; column 3: wild-type; column 4: H272A; column 5: S270A; column 6: S270E. (c) Apoptotic cells in nSMase1 transfectants. Transfected cells were stained with DAPI to assess apoptosis levels. (d) Cell viability in nSMase1 transfectants. Viable cell numbers were determined by using Trypan Blue dye exclusion. (e) The caspase-3 activities in nSMase1 transfectants. Caspase-3 activation was assessed by measuring the hydrolysis of Ac-DEVD-MCA. Each value represents the mean of three independent experiments; the error bars represent the standard deviations. *, p < 0.01 versus mock.
Supplementary Figure 8.
Yabu et al.

## Slide 9
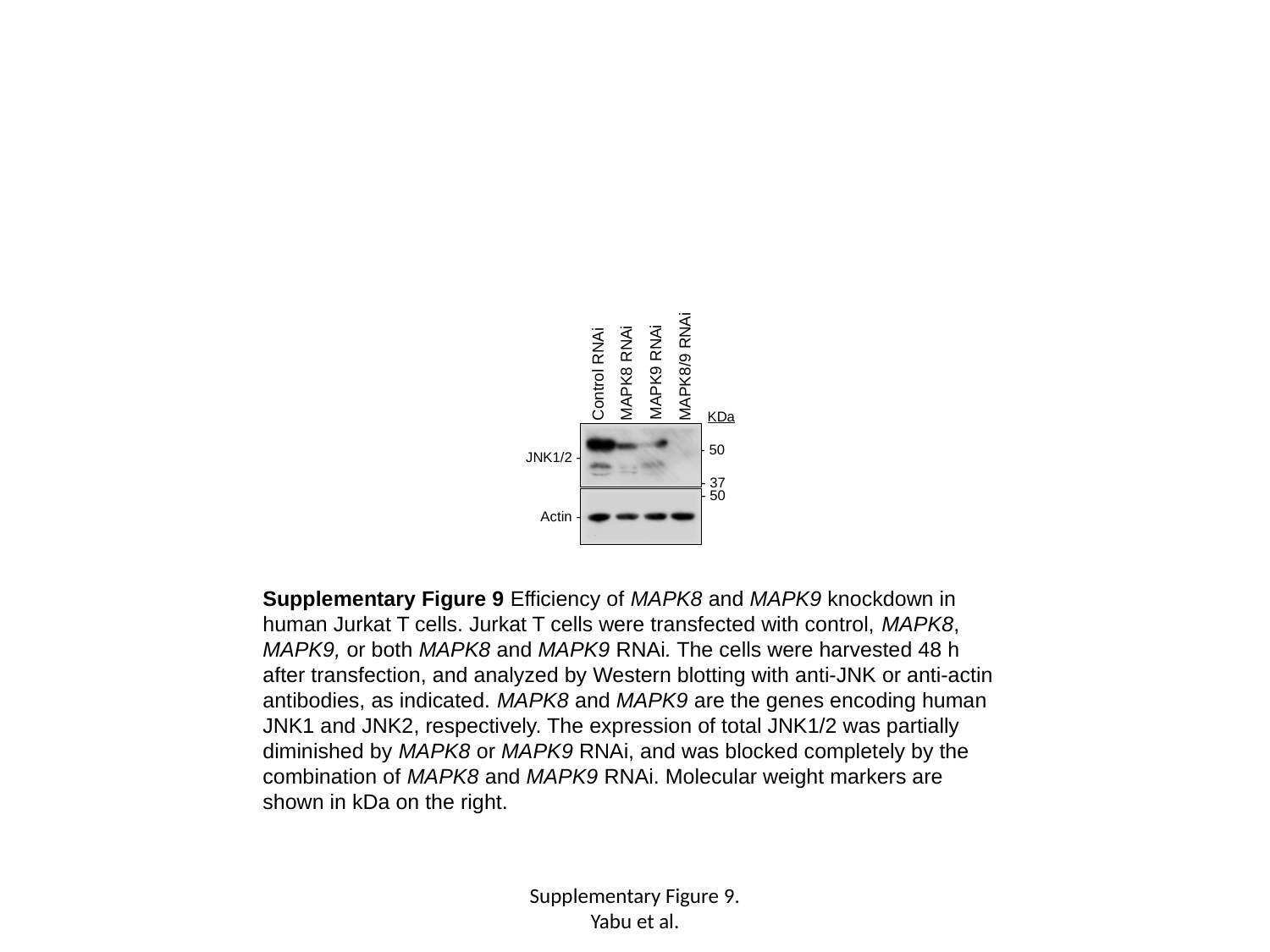

MAPK8/9 RNAi
MAPK9 RNAi
MAPK8 RNAi
Control RNAi
KDa
- 50
JNK1/2 -
- 37
- 50
Actin -
Supplementary Figure 9 Efficiency of MAPK8 and MAPK9 knockdown in human Jurkat T cells. Jurkat T cells were transfected with control, MAPK8, MAPK9, or both MAPK8 and MAPK9 RNAi. The cells were harvested 48 h after transfection, and analyzed by Western blotting with anti-JNK or anti-actin antibodies, as indicated. MAPK8 and MAPK9 are the genes encoding human JNK1 and JNK2, respectively. The expression of total JNK1/2 was partially diminished by MAPK8 or MAPK9 RNAi, and was blocked completely by the combination of MAPK8 and MAPK9 RNAi. Molecular weight markers are shown in kDa on the right.
Supplementary Figure 9.
Yabu et al.

## Slide 10
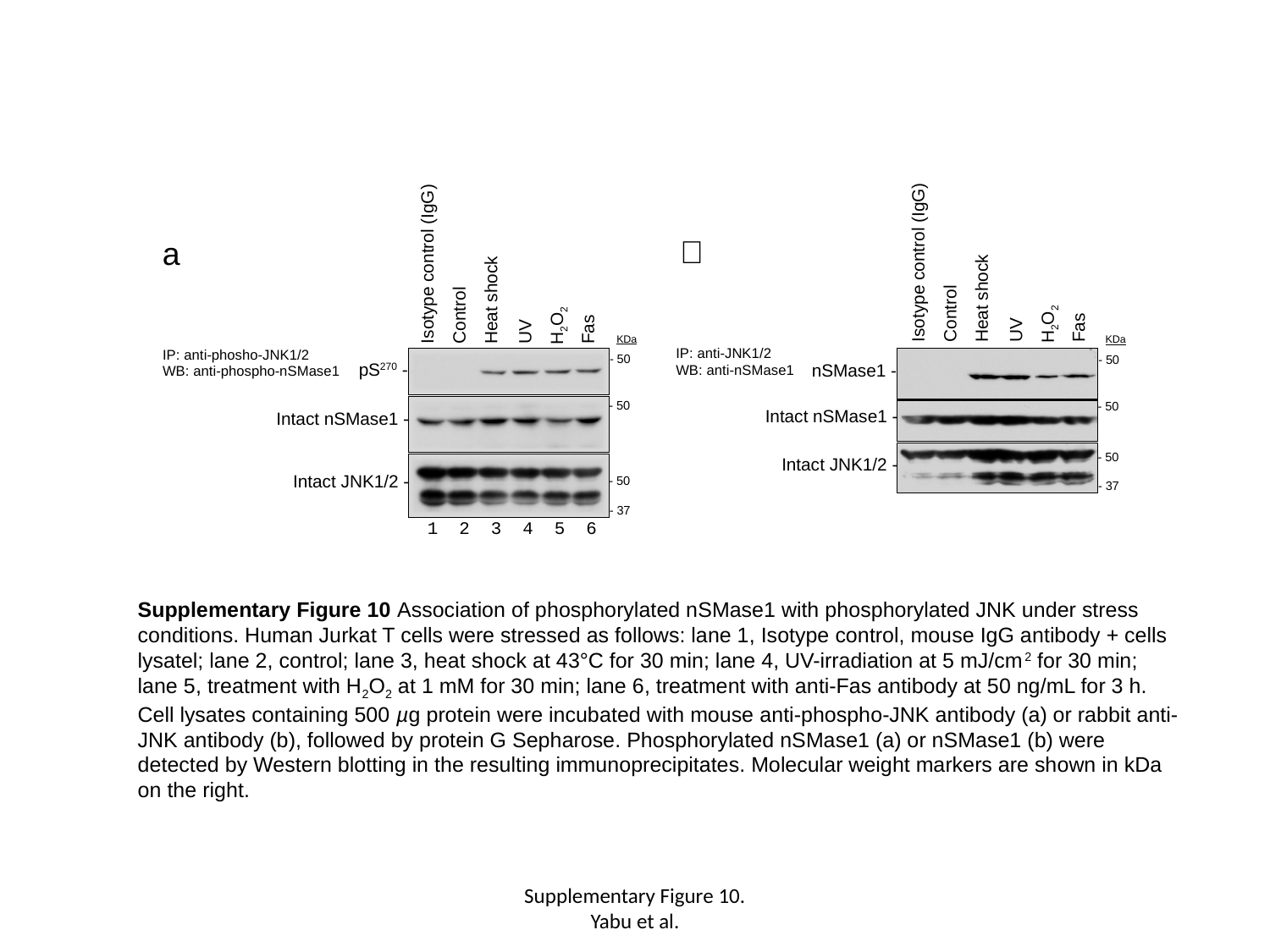

Isotype control (IgG)
Heat shock
Control
H2O2
Fas
UV
KDa
- 50
- 50
- 50
- 37
IP: anti-JNK1/2
WB: anti-nSMase1
 nSMase1 -
Intact nSMase1 -
Intact JNK1/2 -
ｂ
Isotype control (IgG)
Heat shock
Control
H2O2
Fas
UV
KDa
- 50
- 50
- 50
- 37
IP: anti-phosho-JNK1/2
WB: anti-phospho-nSMase1
 pS270 -
Intact nSMase1 -
Intact JNK1/2 -
1 2 3 4 5 6
a
Supplementary Figure 10 Association of phosphorylated nSMase1 with phosphorylated JNK under stress conditions. Human Jurkat T cells were stressed as follows: lane 1, Isotype control, mouse IgG antibody + cells lysatel; lane 2, control; lane 3, heat shock at 43°C for 30 min; lane 4, UV-irradiation at 5 mJ/cm2 for 30 min; lane 5, treatment with H2O2 at 1 mM for 30 min; lane 6, treatment with anti-Fas antibody at 50 ng/mL for 3 h. Cell lysates containing 500 µg protein were incubated with mouse anti-phospho-JNK antibody (a) or rabbit anti-JNK antibody (b), followed by protein G Sepharose. Phosphorylated nSMase1 (a) or nSMase1 (b) were detected by Western blotting in the resulting immunoprecipitates. Molecular weight markers are shown in kDa on the right.
Supplementary Figure 10.
Yabu et al.

## Slide 11
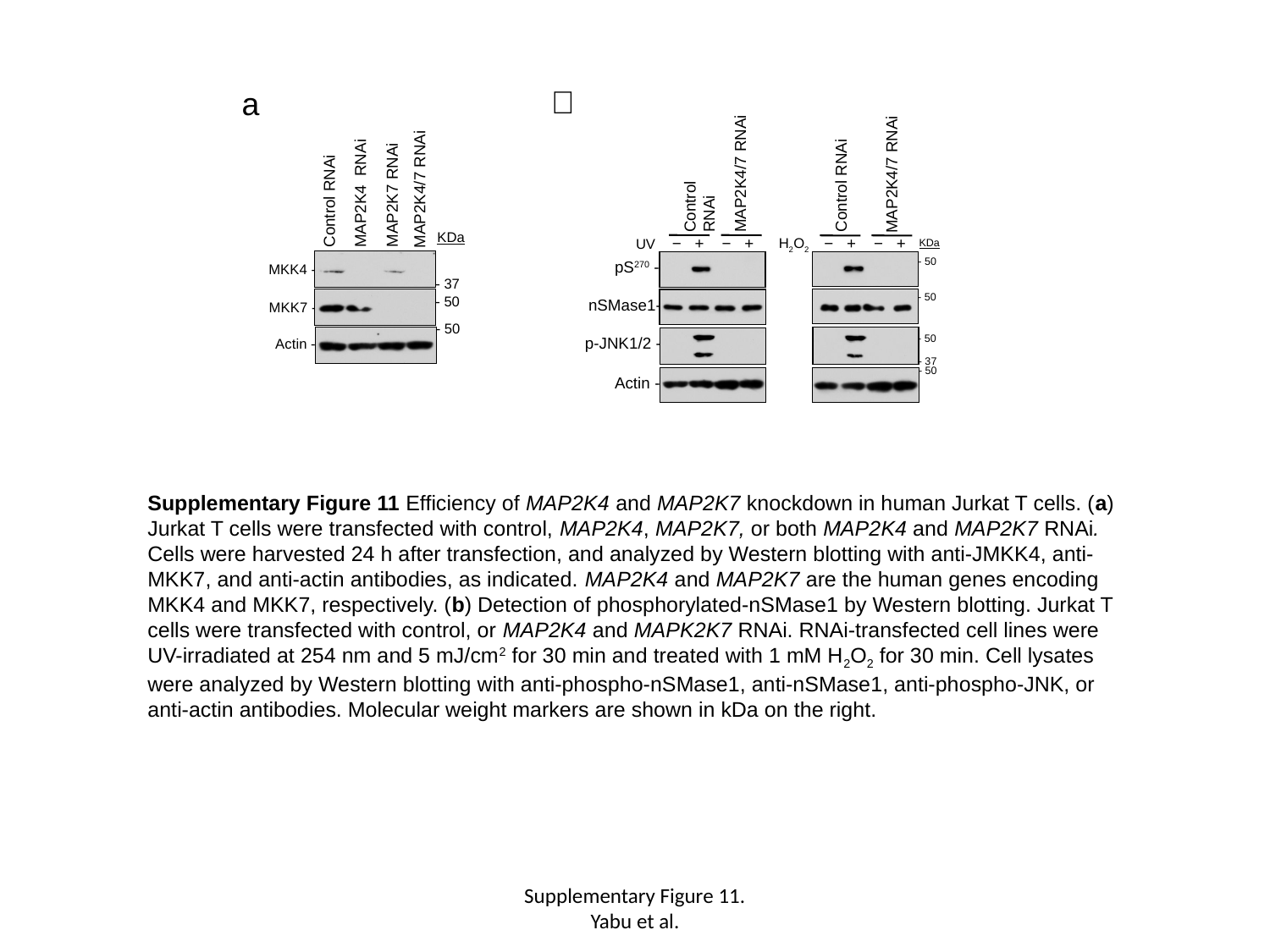

ｂ
a
MAP2K4/7 RNAi
Control RNAi
 − + − +
UV
pS270 -
nSMase1-
p-JNK1/2 -
Actin -
MAP2K4/7 RNAi
Control RNAi
 − + − +
KDa
- 50
- 50
- 50
- 37
- 50
H2O2
MAP2K4/7 RNAi
MAP2K4 RNAi
MAP2K7 RNAi
Control RNAi
KDa
MKK4 -
- 37
- 50
MKK7 -
- 50
Actin -
Supplementary Figure 11 Efficiency of MAP2K4 and MAP2K7 knockdown in human Jurkat T cells. (a) Jurkat T cells were transfected with control, MAP2K4, MAP2K7, or both MAP2K4 and MAP2K7 RNAi. Cells were harvested 24 h after transfection, and analyzed by Western blotting with anti-JMKK4, anti-MKK7, and anti-actin antibodies, as indicated. MAP2K4 and MAP2K7 are the human genes encoding MKK4 and MKK7, respectively. (b) Detection of phosphorylated-nSMase1 by Western blotting. Jurkat T cells were transfected with control, or MAP2K4 and MAPK2K7 RNAi. RNAi-transfected cell lines were UV-irradiated at 254 nm and 5 mJ/cm2 for 30 min and treated with 1 mM H2O2 for 30 min. Cell lysates were analyzed by Western blotting with anti-phospho-nSMase1, anti-nSMase1, anti-phospho-JNK, or anti-actin antibodies. Molecular weight markers are shown in kDa on the right.
Supplementary Figure 11.
Yabu et al.

## Slide 12
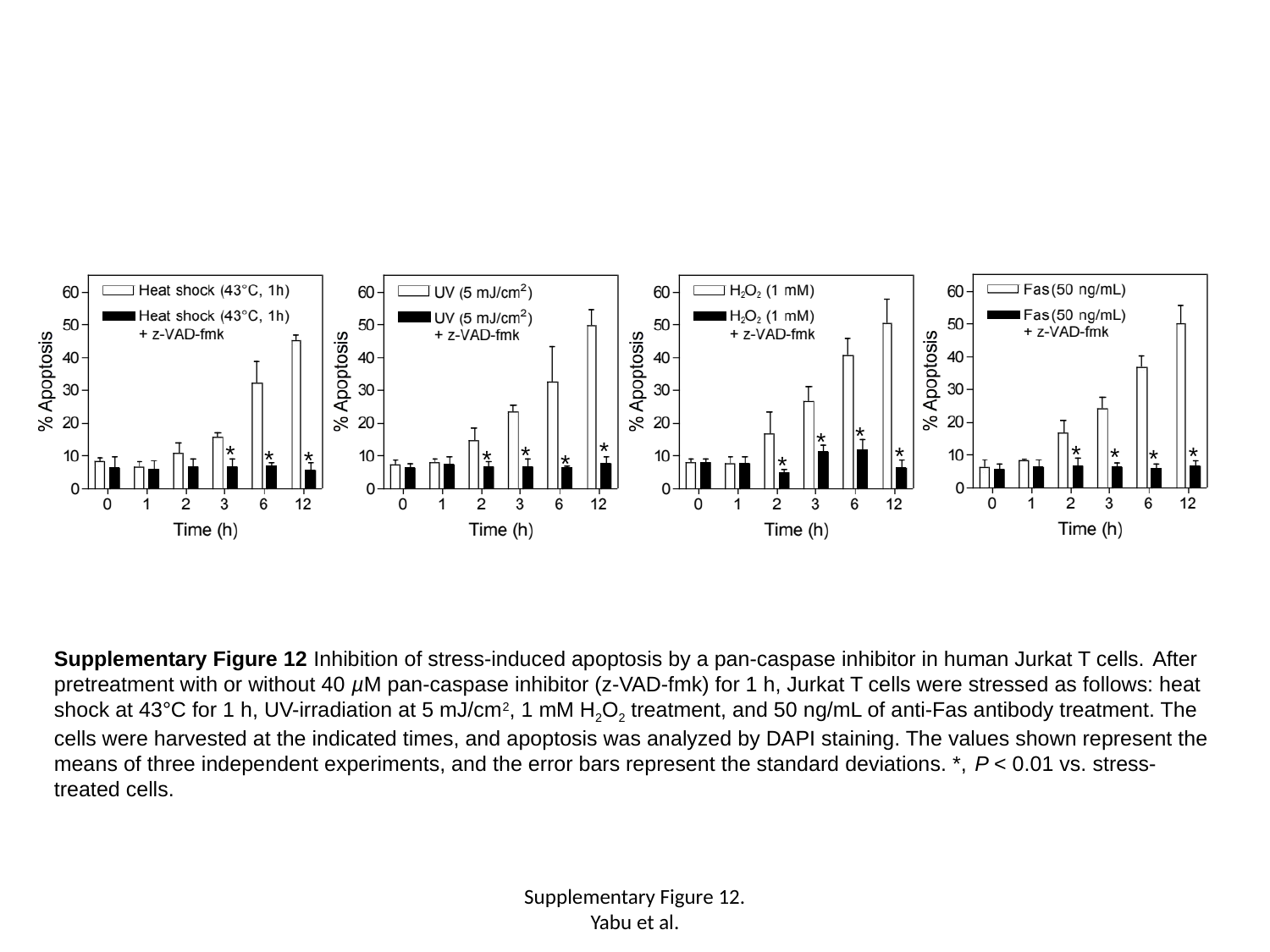

*
*
*
*
*
*
*
*
*
*
*
*
*
*
*
Supplementary Figure 12 Inhibition of stress-induced apoptosis by a pan-caspase inhibitor in human Jurkat T cells. After pretreatment with or without 40 µM pan-caspase inhibitor (z-VAD-fmk) for 1 h, Jurkat T cells were stressed as follows: heat shock at 43°C for 1 h, UV-irradiation at 5 mJ/cm2, 1 mM H2O2 treatment, and 50 ng/mL of anti-Fas antibody treatment. The cells were harvested at the indicated times, and apoptosis was analyzed by DAPI staining. The values shown represent the means of three independent experiments, and the error bars represent the standard deviations. *, P < 0.01 vs. stress-treated cells.
Supplementary Figure 12.
Yabu et al.

## Slide 13
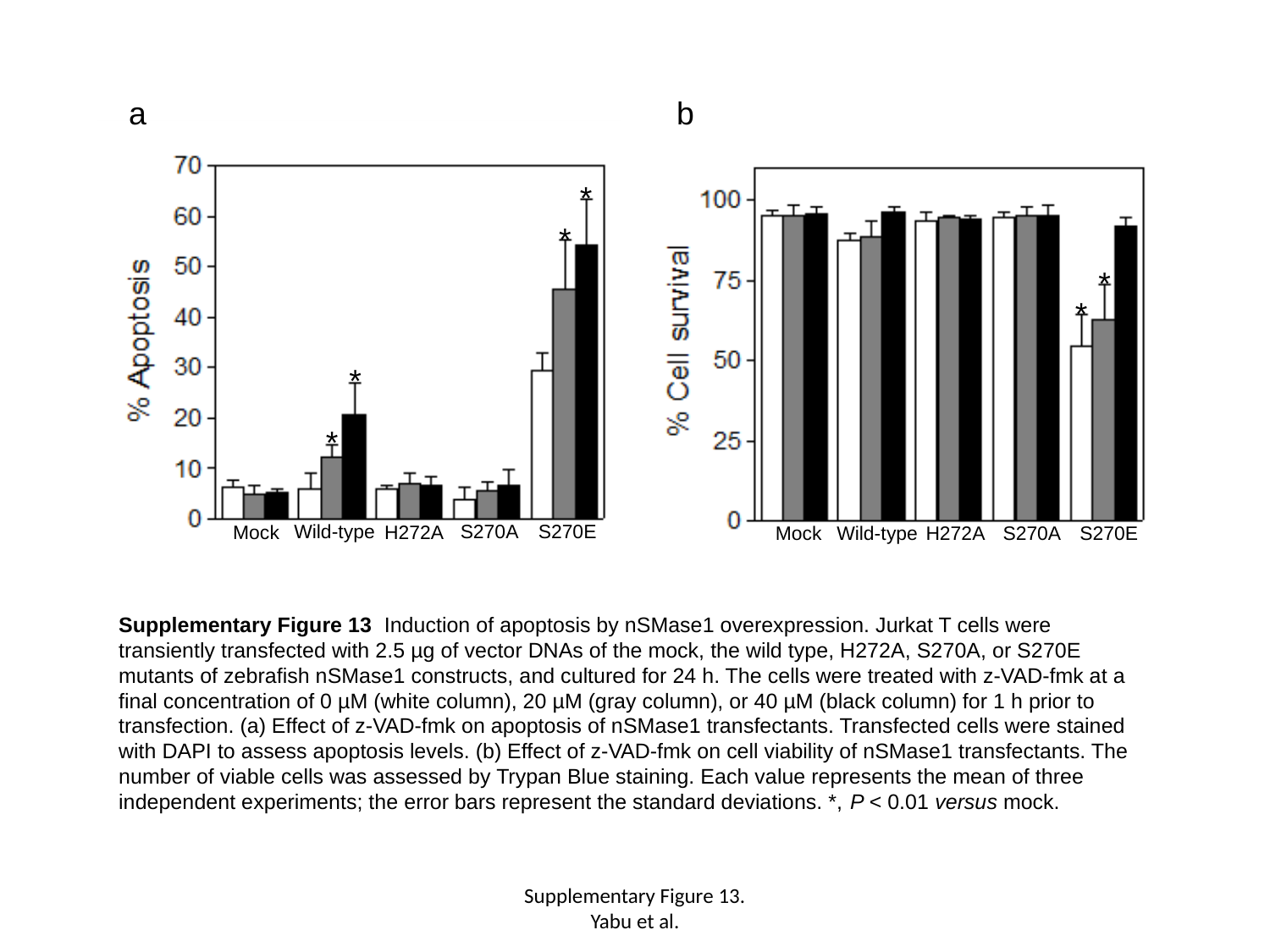

a
*
*
*
*
S270E
S270A
Wild-type
Mock
H272A
b
*
*
S270E
S270A
Wild-type
Mock
H272A
Supplementary Figure 13 Induction of apoptosis by nSMase1 overexpression. Jurkat T cells were transiently transfected with 2.5 µg of vector DNAs of the mock, the wild type, H272A, S270A, or S270E mutants of zebrafish nSMase1 constructs, and cultured for 24 h. The cells were treated with z-VAD-fmk at a final concentration of 0 µM (white column), 20 µM (gray column), or 40 µM (black column) for 1 h prior to transfection. (a) Effect of z-VAD-fmk on apoptosis of nSMase1 transfectants. Transfected cells were stained with DAPI to assess apoptosis levels. (b) Effect of z-VAD-fmk on cell viability of nSMase1 transfectants. The number of viable cells was assessed by Trypan Blue staining. Each value represents the mean of three independent experiments; the error bars represent the standard deviations. *, P < 0.01 versus mock.
Supplementary Figure 13.
Yabu et al.

## Slide 14
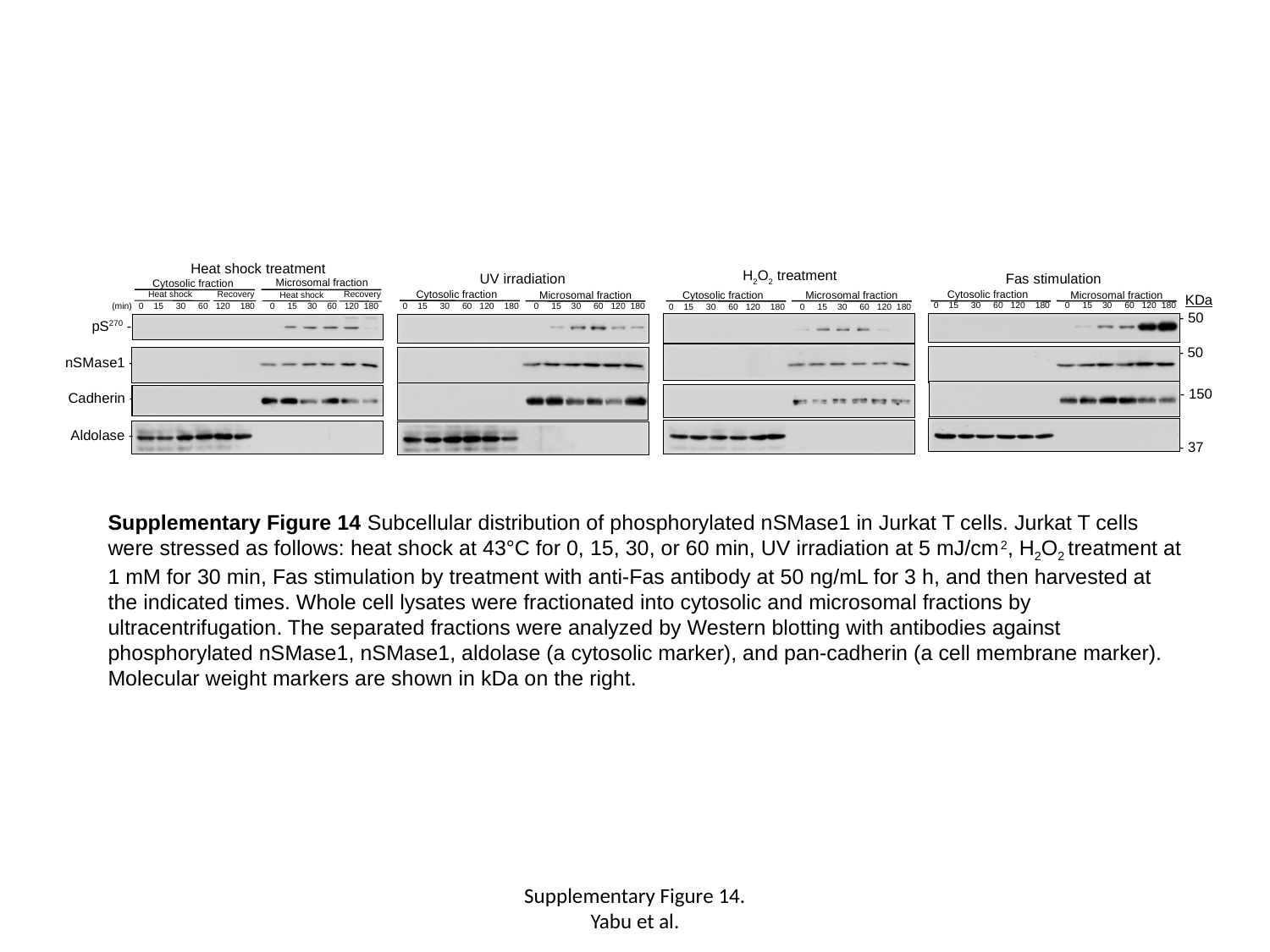

Heat shock treatment
Microsomal fraction
Cytosolic fraction
Recovery
Heat shock
Recovery
Heat shock
(min)
0 15 30 60 120 180 0 15 30 60 120 180
pS270 -
nSMase1 -
Cadherin -
Aldolase -
H2O2 treatment
Cytosolic fraction
Microsomal fraction
0 15 30 60 120 180 0 15 30 60 120 180
UV irradiation
Cytosolic fraction
Microsomal fraction
0 15 30 60 120 180 0 15 30 60 120 180
Fas stimulation
Cytosolic fraction
Microsomal fraction
KDa
0 15 30 60 120 180 0 15 30 60 120 180
- 50
- 50
- 150
- 37
Supplementary Figure 14 Subcellular distribution of phosphorylated nSMase1 in Jurkat T cells. Jurkat T cells were stressed as follows: heat shock at 43°C for 0, 15, 30, or 60 min, UV irradiation at 5 mJ/cm2, H2O2 treatment at 1 mM for 30 min, Fas stimulation by treatment with anti-Fas antibody at 50 ng/mL for 3 h, and then harvested at the indicated times. Whole cell lysates were fractionated into cytosolic and microsomal fractions by ultracentrifugation. The separated fractions were analyzed by Western blotting with antibodies against phosphorylated nSMase1, nSMase1, aldolase (a cytosolic marker), and pan-cadherin (a cell membrane marker). Molecular weight markers are shown in kDa on the right.
Supplementary Figure 14.
Yabu et al.

## Slide 15
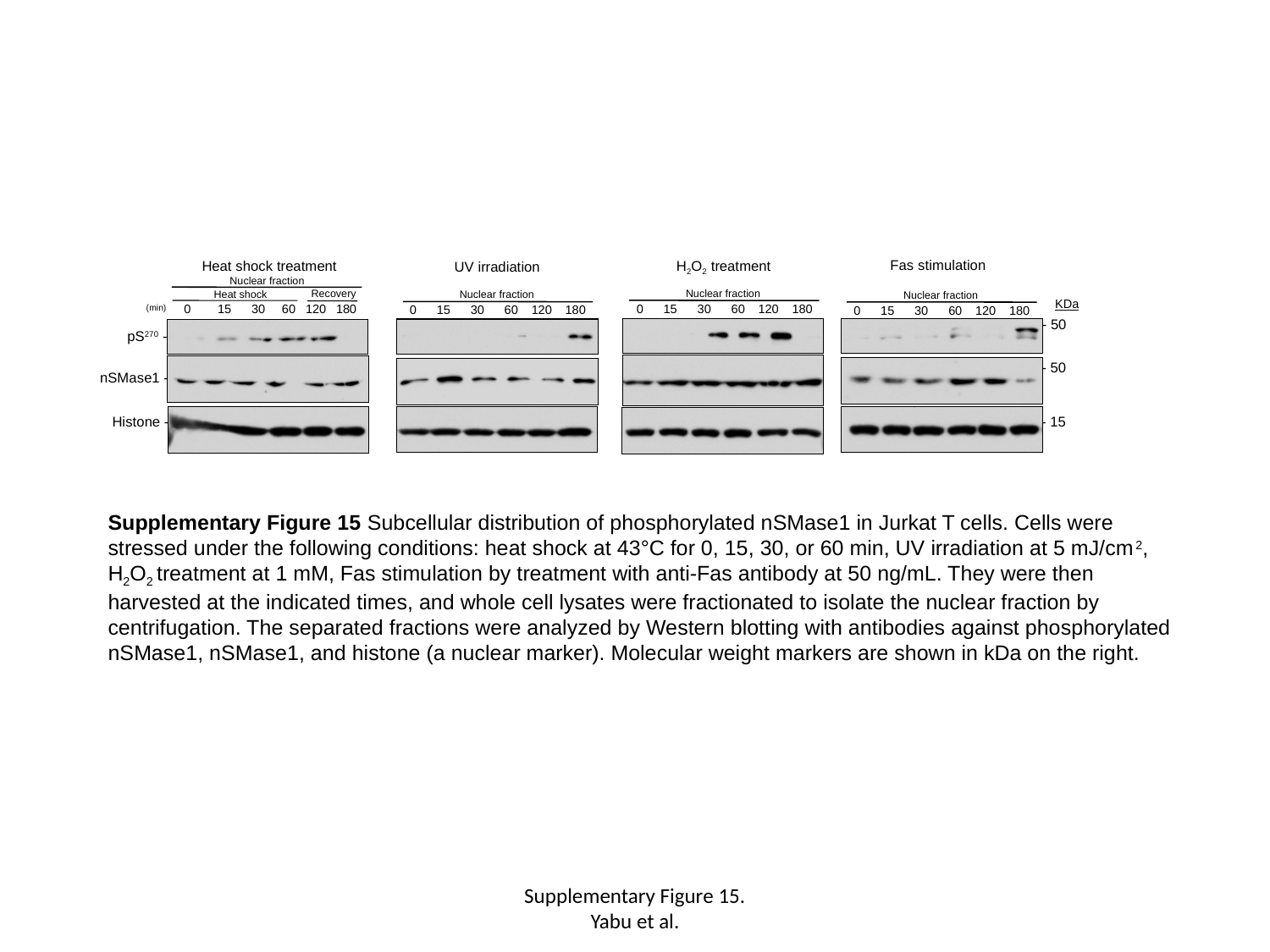

Fas stimulation
Nuclear fraction
0 15 30 60 120 180
KDa
- 50
- 50
- 15
Heat shock treatment
Nuclear fraction
Recovery
Heat shock
0 15 30 60 120 180
(min)
pS270 -
nSMase1 -
Histone -
H2O2 treatment
Nuclear fraction
0 15 30 60 120 180
UV irradiation
Nuclear fraction
0 15 30 60 120 180
Supplementary Figure 15 Subcellular distribution of phosphorylated nSMase1 in Jurkat T cells. Cells were stressed under the following conditions: heat shock at 43°C for 0, 15, 30, or 60 min, UV irradiation at 5 mJ/cm2, H2O2 treatment at 1 mM, Fas stimulation by treatment with anti-Fas antibody at 50 ng/mL. They were then harvested at the indicated times, and whole cell lysates were fractionated to isolate the nuclear fraction by centrifugation. The separated fractions were analyzed by Western blotting with antibodies against phosphorylated nSMase1, nSMase1, and histone (a nuclear marker). Molecular weight markers are shown in kDa on the right.
Supplementary Figure 15.
Yabu et al.

## Slide 16
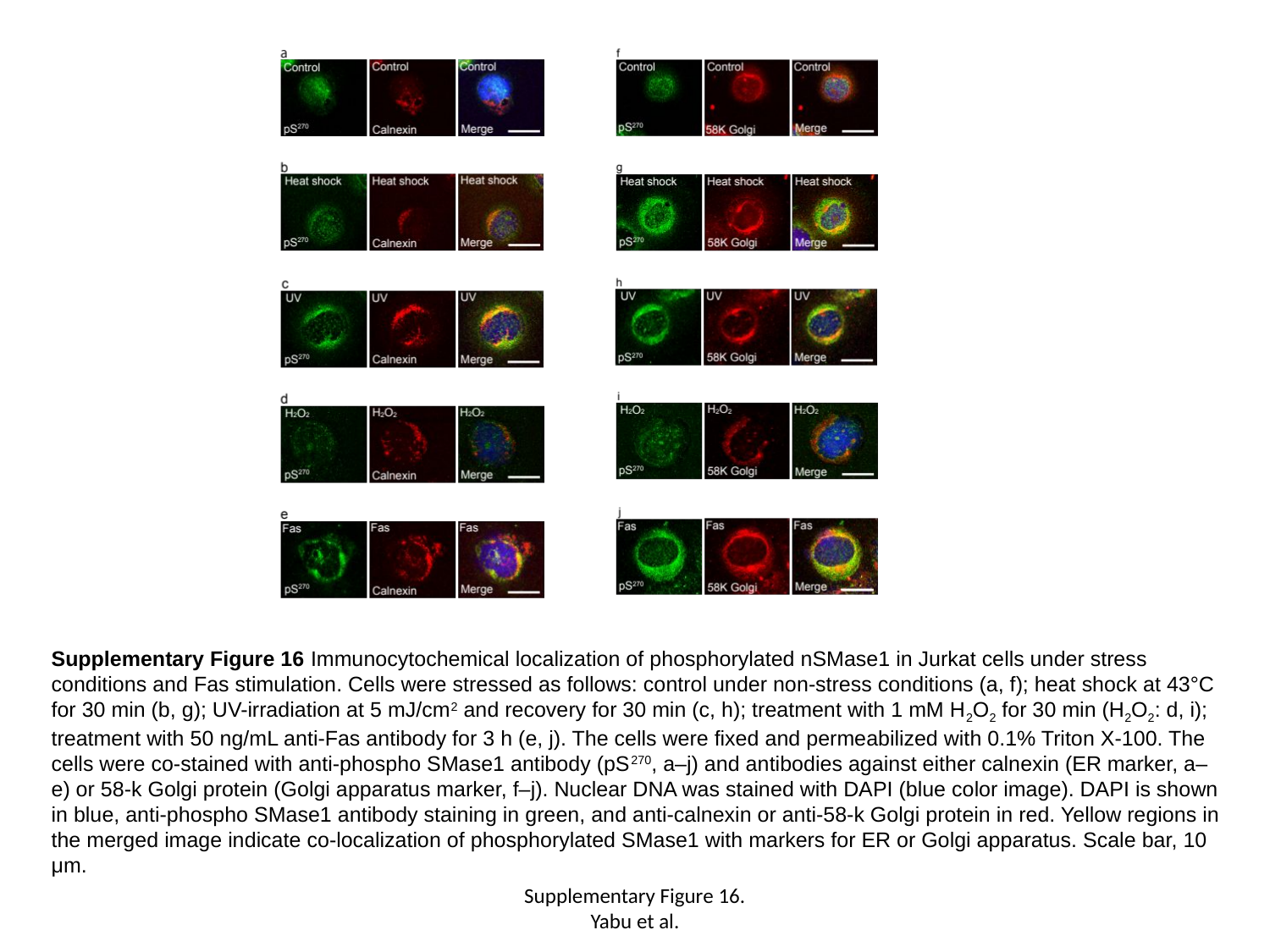

Supplementary Figure 16 Immunocytochemical localization of phosphorylated nSMase1 in Jurkat cells under stress conditions and Fas stimulation. Cells were stressed as follows: control under non-stress conditions (a, f); heat shock at 43°C for 30 min (b, g); UV-irradiation at 5 mJ/cm2 and recovery for 30 min (c, h); treatment with 1 mM H2O2 for 30 min (H2O2: d, i); treatment with 50 ng/mL anti-Fas antibody for 3 h (e, j). The cells were fixed and permeabilized with 0.1% Triton X-100. The cells were co-stained with anti-phospho SMase1 antibody (pS270, a–j) and antibodies against either calnexin (ER marker, a–e) or 58-k Golgi protein (Golgi apparatus marker, f–j). Nuclear DNA was stained with DAPI (blue color image). DAPI is shown in blue, anti-phospho SMase1 antibody staining in green, and anti-calnexin or anti-58-k Golgi protein in red. Yellow regions in the merged image indicate co-localization of phosphorylated SMase1 with markers for ER or Golgi apparatus. Scale bar, 10 μm.
Supplementary Figure 16.
Yabu et al.
